# Conformal Invariance of Boundary Touching Loops of FK Ising Model

**DOI:** 10.1007/s00220-019-03437-0

**Published:** 2019-05-30

**Authors:** Antti Kemppainen, Stanislav Smirnov

**Affiliations:** 1 Department of Mathematics and Statistics, University of Helsinki, P.O. Box 68, 00014 Helsinki, Finland; 2 Section de mathématiques, Université de Genève, 2-4, rue du Lièvre, c.p. 64, 1211 Geneva 4, Switzerland; 3 Chebyshev Laboratory, St. Petersburg State University, St. Petersburg, Russia; 4 Skolkovo Institute of Science and Technology, Moscow, Russia

## Abstract

In this article we show the convergence of a loop ensemble of interfaces in the FK Ising model at criticality, as the lattice mesh tends to zero, to a unique conformally invariant scaling limit. The discrete loop ensemble is described by a canonical tree glued from the interfaces, which then is shown to converge to a tree of branching SLEs. The loop ensemble contains unboundedly many loops and hence our result describes the joint law of infinitely many loops in terms of SLE type processes, and the result gives the full scaling limit of the FK Ising model in the sense of random geometry of the interfaces. Some other results in this article are convergence of the exploration process of the loop ensemble (or the branch of the exploration tree) to $\hbox {SLE}(\kappa ,\kappa -6)$, $\kappa =16/3$, and convergence of a generalization of this process for 4 marked points to $\hbox {SLE}[\kappa ,Z]$, $\kappa =16/3$, where *Z* refers to a partition function. The latter SLE process is a process that can’t be written as a $\hbox {SLE}(\kappa ,\rho _1,\rho _2,\ldots )$ process, which are the most commonly considered generalizations of SLEs.

## Introduction

### The setup informally

**Fig. 1:**
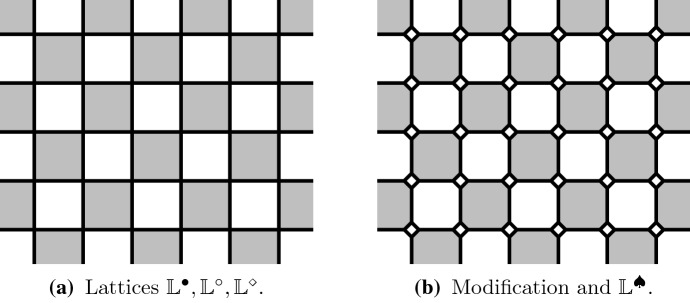
The square lattices we are considering are $\mathbb {L}^\bullet$ formed by the centers of the black squares (the vertices are connected by an edge if the corresponding squares touch by corners), $\mathbb {L}^\circ$ formed by the centers of the white squares (the edges similarly as for $\mathbb {L}^\bullet$) and $\mathbb {L}^\diamond$ formed by the vertices and edges of the black (or equivalently white) squares. We will also consider the square–octagon lattice $\mathbb {L}^\spadesuit$ (infinite graph formed by the vertices and edges of the squares and octagons in the picture) which we see as a modification of $\mathbb {L}^\diamond$

The *Ising model* is one of the most studied lattice models in statistical physics. The Ising model (and Potts models generalizing it) have percolation-type representations called *Fortuin–Kasteleyn random cluster models* (FK model). Given a graph the Ising model assigns probabilities to configurations of $\pm 1$ spins on the vertices of the graph and the random cluster model assigns probabilities to configurations of open/closed edges. FK model is obtained from the percolation model (giving weight *p* to each open edge and $1-p$ to each closed edge) by weighting by a factor *q* per each connected cluster of open edges. We will consider the FK Ising model, i.e., FK model whose parameter value *q* corresponds to the Ising model, on a square lattice. The lattice we are considering is the usual square lattice $\mathbb {Z}^2$, for convenience rotated by $45^\circ$. In Fig. 1a it is the lattice formed by the centers of the black squares.

**Fig. 2:**
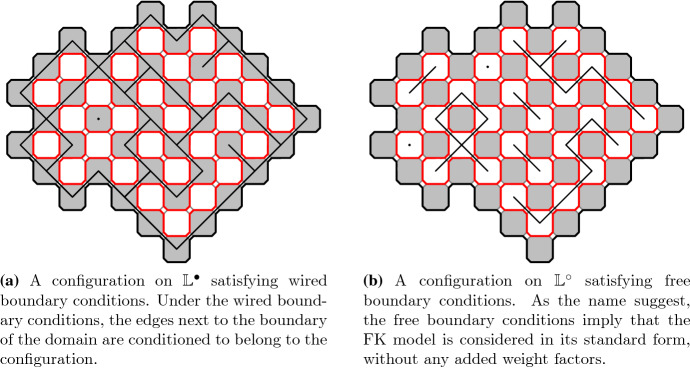
FK configurations with wired and free boundary conditions on graphs dual to each other. Notice that these two configurations are dual to each other and thus have the same loop representations

We will consider a bounded simply connected subgraph of the square lattice. We will make this definition clearer later. An FK configuration in the graph is illustrated in Fig. 2a and its dual configuration in Fig. 2b. The dual configuration is defined on the dual lattice, formed by the centers of the white squares in Fig. 1a, with the rule that exactly one of any (primal) edge and its dual edge (the dual edge is the unique edge on the dual lattice crossing the primal edge) is present in the configuration or in the dual configuration. The so called *loop representation* of the FK model is defined on so called *medial lattice*, shown in Fig. 1a and formed by the corners of the squares, by taking the inner and outer boundaries of all the connected components in a configuration of edges. The loop representation can be seen as a dense collection of *simple* loops when we resolve the possible double vertices by the modification illustrated in Fig. 1b or Fig. 2.

The duality of the FK configurations described above gives a mapping between the set of configurations with wired boundary conditions and the set of configurations with free boundary conditions, defined on the graph and on its dual, respectively. One can check that the FK model parameter values *p* and *q* get mapped under this involution of FK configurations to values $p^*$ and $q^*=q$ where $p^*$ is given by

1 $$\begin{aligned} \frac{p \, p^*}{(1- p)(1- p^*)} = q . \end{aligned}$$

In this article we will consider critical point of the model which happens to be the self-dual value of *p*, that is, $p=p_c$ when $p=p^*$. For the FK Ising model, the parameter $q=2$ and the *critical parameter*$p_c= \frac{\sqrt{2}}{1+\sqrt{2}}$. By the fact that it is the self-dual point we see that in Fig. 2a, b the FK models have the same parameter values. The only difference is in boundary conditions. The resulting loop configurations have the same law for the critical parameter on both setups, either wired boundary conditions on the primal graph or free boundary conditions on the dual graph.

The correspondence of boundary conditions is slightly more complicated for other boundary conditions than the wired and free ones.

#### The role of the critical parameter.

The parameter is chosen to be critical for several reasons. First of all it is expected that only for this value of the parameter the scaling limit will be non-trivial. For the other values, we get either zero or one macroscopic loop and all the other loops will be microscopic and vanishing in the scaling limit. The only macroscopic loop will be rather uninteresting as it will follow closely the boundary, so that the loop will fluctuate from the boundary only to a distance which vanishes in the scaling limit. In contrast, for the critical parameter the scaling limit will consist of (countably) infinite number of loops as we will be showing.

The second reason for selecting this value of the parameter is more technical. For that value, the observable we are defining in Sect. 4.1 is going to satisfy a relation which we can interpret as a discrete version of the Cauchy–Riemann equations. This makes it possible to pass to the limit and recover in the limit a holomorphic function solving a boundary value problem.

The third reason for the choice of the critical parameter is that at criticality we expect that the loop collection will have a conformal symmetry. This is already suggested by the existence of the holomorphic observable.

In fact, the discrete holomorphicity and related techniques allow us to control the scaling limit well and to identify the scaling limit and to show its conformal invariance. And thus can be seen as the most important reason to consider a system at criticality.

**Fig. 3:**
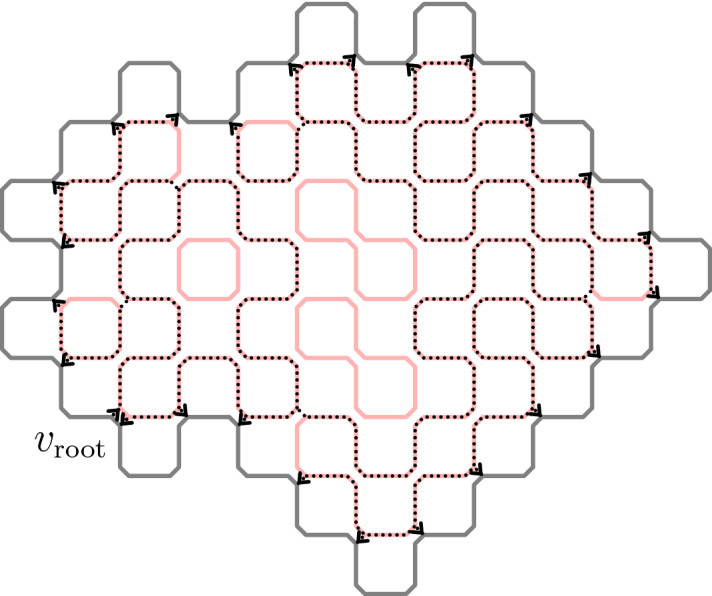
The loop collection and the exploration tree: solid  (including solid pink on the background of dotted lines) indicate the loops. The dotted black lines form the tree. The dotted black lines with no pink on background are the jumps from one loop to another

### The exploration tree and the main results

#### The exploration tree.

Figure 3 illustrates the construction of the *exploration tree* of a loop configuration. The (chordal) exploration tree connects the fixed root vertex to any other boundary point. The construction of the branch form the root to a fixed target vertex is the following:

1. To initialize the process cut open the loop next to the root vertex and *start the process* at that location on that loop.
2. *Explore the current loop in clockwise direction* until the target point is reached or any point at which it is clear that it is impossible to reach the target point along the current loop (meaning that the current location of the exploration is disconnected from the target in the discrete slit domain where the explored part has been removed from the original domain).
3. *If the target was reached, then stop and return* (as the result of the algorithm) *the path* concatenated from the subpaths of loops in the order that they were explored.
4. *If the target was not reached yet, cut open the loop next to the current location* and jump to that loop and *go to Step (2)*.

The construction depends on the direction of the exploration which was chosen in (2) to be clockwise. Instead of a deterministic choice, independent coin flips could be used to decide whether to follow each loop in clockwise or counterclockwise direction. This would lead to a different process. In this article we will use the above construction which suits well our purposes. After all, the main goal is to show convergence of the loop collection, and the above construction agrees well with our observable.

#### Main result.

The main theorem of this article is the following result. For its formulation, call a discrete domain *admissible* if it is simply connected and bounded and its boundary consists of a chain of black octagons and small squares as in Fig. 2a. More generally, introduce lattice mesh by scaling the lattices ($\mathbb {L}^\bullet , \mathbb {L}^\circ , \mathbb {L}^\diamond , \mathbb {L}^\spadesuit$ etc.) by a factor $\delta >0$ and consider discrete, admissible domains with lattice mesh $\delta >0$, and use notation $\Omega ^{(\delta )}$ to explicitly refer to such a domain. A sequence of simply connected domains $\Omega _n$ (say $\Omega _n=\Omega ^{(\delta _n)}$) converges to a domain $\Omega$ in *Carathéodory sense* with respect to a point $w_0\in \Omega$, if $w_0\in \Omega _n$ for all *n* and the conformal and onto maps $\psi _n: \mathbb {D}\rightarrow \Omega _n$ with $\psi _n(0)=w_0$ and $\psi _n^{\prime }(0)>0$ converge uniformly on compact subsets of $\mathbb {D}$ to a conformal and onto map $\psi : \mathbb {D}\rightarrow \Omega$ with $\psi (0)=w_0$ and $\psi ^{\prime }(0)>w_0$. Notice that then the inverse maps $\phi _n = \psi ^{-1}_n$ converge uniformly on any compact subset *K* of $\Omega$ (*K* belongs to the domain of $\phi _n$ for large *n*).

##### Theorem 1.1

Let $\Omega _{\delta _n}$ be a sequence of admissible domains that converges to a domain $\Omega$ in Carathéodory sense with respect to some fixed $w_0\in \Omega$ and let $\Theta _{\partial ,\delta _n}$ be the random collection of loops which is the collection of all loops of the loop representation of the FK Ising model on $\Omega _{\delta _n}$ that intersect the boundary (the boundary touching loops). Let $\phi _n$ be a conformal map that sends $\Omega _n$ onto the unit disc and $w_0$ to 0. Then as $n\rightarrow \infty$, the sequence of random loop collections $\phi _n(\Theta _{\partial ,\delta _n})$ converges weakly to a limit $\Theta$ whose law is independent of the choices of $\Omega , \delta _n, \Omega _{\delta _n}, w_0$ and $\phi _n$, and hence the law is invariant under all conformal isomorphisms of $\mathbb {D}$.

Moreover the law of $\Theta$ is the boundary touching loops of $\hbox {CLE}(\kappa )$ with $\kappa =16/3$ and is given by the image of the $\hbox {SLE}(\kappa ,\kappa -6)$ exploration tree with $\kappa =16/3$ under a tree-to-loops mapping which inverts the construction in Sect. 1.2.1 and is explained in more details in Sect. 2.3 (including definitions needed for understanding this theorem and making the statements more precise).

##### Remark 1.2

In this article we will prove Theorem 1.1 only in the case that $\Omega$ is smooth and that each of its discrete approximations have boundary which close to the boundary of $\Omega$ in the sense that their distance is bounded by a uniform constant times the lattice step of the approximation. By these assumptions, we will exclude the cases where the boundary forms long fjords to have better estimates for the harmonic measure. The general case follows from the sequel [15] of this article on the *radial* exploration tree of the FK Ising model. These restrictions are technical and they are not needed, for instance, for the convergence in the so-called 4-point case (Sect. 4.4.1 and other arguments leading to the convergence of the interface to $\hbox {SLE}[\kappa ,Z]$ in Sect. 5.5).

The present article aims to provide clear details for the basic proof techniques which include the regularity properties of trees, derivation of the martingale observables and the corresponding martingale characterization in the boundary-touching-loop setting. In principle, one should be able to deduce the complete picture by repeatedly iterating this construction inside the resulting holes appearing after removing the boundary touching loops, the main difficult point being the fractal boundary. Instead, in the sequel [15] we build the complete tree towards interior points, thus not having to deal with fractal boundaries. This requires working with a more complicated observable, and the proof in the current article better explains what follows in the sequel.

Our result in the 4-point setting is interesting in its own right. In a follow-up paper [16], we use it to show that the interface conditioned on an internal arc pattern converges towards so-called hypergeometric SLE.

**Fig. 4:**
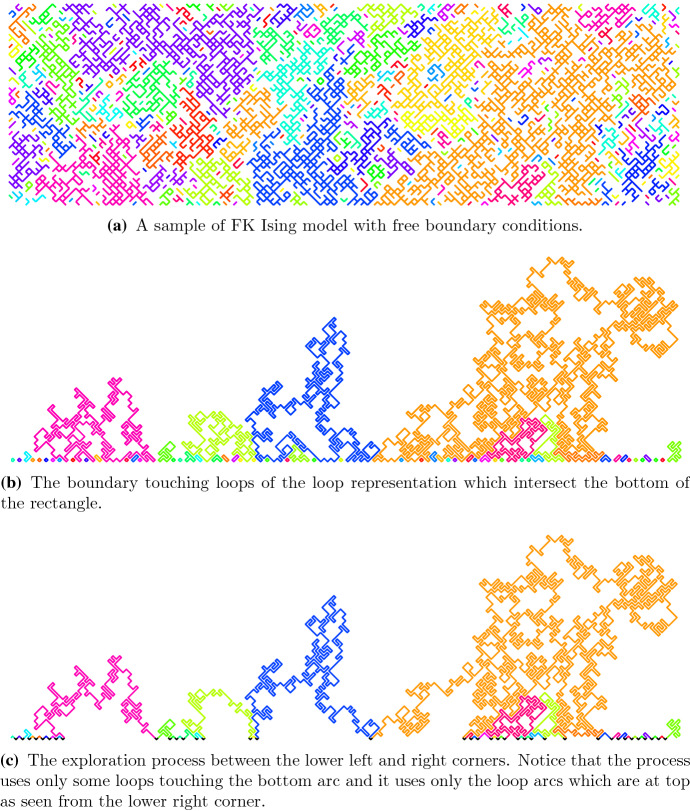
A sample configuration of the critical FK Ising model on a rectangular domain with free boundary conditions. Components with only one vertex are not shown. The colors distinguish different components. Notice that in this particular sample, the large orange loop happens to come fairly close to the boundary without disconnecting the small loops to its right. Thus the exploration path turns and explores those boundary touching loops inside the “fjord”

A sample of FK Ising branch is illustrated in Fig. 4.

### Previous results on conformally invariant scaling limits of random curves and loops

So far, convergence of a single discrete interface to $\hbox {SLE}(\kappa )$’s has been established for but a few models: $\kappa =2$ and $\kappa =8$ [17], $\kappa =3$ and $\kappa =\frac{16}{3}$ [8], $\kappa =4$ [21, 22] and $\kappa =6$ [24, 25]. However, the framework for the full scaling limit, including all interfaces, is less developed: in addition to the present article only $\kappa =3$ [4] and $\kappa =6$ [6] and our subsequent work [15]. A similar result on a collection of random curves is the convergece of the free arc ensemble of the Ising model in [3].

### Organization of the article

We will give further definitions in Sect. 2. In Sect. 3 we explore the regularity and tightness properties of the loop configurations and the exploration trees based on crossing estimates. This gives a priori knowledge needed in the main argument. In Sect. 4 we define the holomorphic observable and show its convergence. In Sect. 5 we combine these tools and extract information from the observable so that we can characterize the scaling limit and prove the main theorem in Sect. 6.

## The Setup and More Details of the Main Result

### Graph theoretical notations and setup

In this article the lattice $\mathbb {L}^\bullet$ is the square lattice $\mathbb {Z}^2$ rotated by $\pi /4$, $\mathbb {L}^\circ$ is its dual lattice, which itself is also a square lattice and $\mathbb {L}^\diamond$ is their (common) medial lattice. More specifically, we define three lattices $G=(V(G),E(G))$, where $G= \mathbb {L}^\bullet ,\mathbb {L}^\circ ,\mathbb {L}^\diamond$, as

2 $$\begin{aligned} V( \mathbb {L}^\bullet )= & {} \left\{ (i,j) \in \mathbb {Z}^2: i+j \text { even} \right\} , \quad E( \mathbb {L}^\bullet ) = \left\{ \{v,w\} \subset V( \mathbb {L}^\bullet ) \,:\, |v-w| = \sqrt{2} \right\} , \nonumber \\ \end{aligned}$$

3 $$\begin{aligned} V( \mathbb {L}^\circ )= & {} \left\{ (i,j) \in \mathbb {Z}^2 : i+j \text { odd} \right\} , \quad E( \mathbb {L}^\circ ) = \left\{ \{v,w\} \subset V( \mathbb {L}^\circ ) \,:\, |v-w| = \sqrt{2} \right\} , \nonumber \\ \end{aligned}$$

4 $$\begin{aligned} V( \mathbb {L}^\diamond )= & {} (1/2 + \mathbb {Z})^2 , \quad E( \mathbb {L}^\diamond ) = \left\{ \{v,w\} \subset V( \mathbb {L}^\diamond ) : |v-w| = 1\right\} . \end{aligned}$$

Notice that sites of $\mathbb {L}^\diamond$ are the midpoints of the edges of $\mathbb {L}^\bullet$ and $\mathbb {L}^\circ$.

We call the vertices and edges of $V( \mathbb {L}^\bullet )$*black* and the vertices and edges of $V( \mathbb {L}^\circ )$*white*. Correspondingly the faces of $\mathbb {L}^\diamond$ are colored black and white depending whether the center of that face belongs to $V( \mathbb {L}^\bullet )$ or $V( \mathbb {L}^\circ )$.

The directed version $\mathbb {L}^\diamond _\rightarrow$ is defined by setting $V(\mathbb {L}^\diamond _\rightarrow )=V(\mathbb {L}^\diamond )$ and orienting the edges around any black face in the counter-clockwise direction.

The modified medial lattice $\mathbb {L}^\spadesuit$, which is a square–octagon lattice, is obtained from $\mathbb {L}^\diamond$ by replacing each site by a small square. See Fig. 1b. The oriented lattice $\mathbb {L}^\spadesuit _\rightarrow$ is obtained from $\mathbb {L}^\spadesuit$ by orienting the edges around black and white octagonal faces in counter-clockwise and clockwise directions, respectively.

#### Definition 2.1

A simply connected, non-empty, bounded domain $\Omega$ is said to be a *wired*$\mathbb {L}^\spadesuit _\rightarrow$-*domain* (or *admissible domain*) if $\partial \Omega$ oriented in counter-clockwise direction is a path in $\mathbb {L}^\spadesuit _\rightarrow$.

**Fig. 5:**
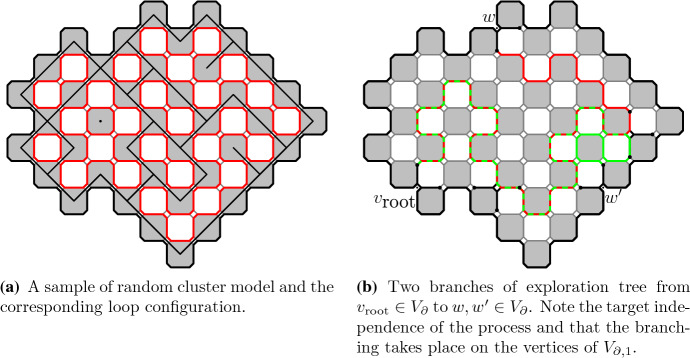
The construction of the exploration tree of the random cluster model

See Fig. 5 for an example of such a domain. The wired $\mathbb {L}^\spadesuit _\rightarrow$-domains are in one to one correspondence with non-empty finite subgraphs of $\mathbb {L}^\bullet$ which are simply connected, i.e., they are graphs who have an unique unbounded face and the rest of the faces are unit-size squares.

### FK Ising model: notations and setup for the full scaling limit

Let *G* be a simply connected subgraph of the square lattice $\mathbb {L}^\bullet$. Consider the random cluster measure $\mu =\mu _{p,q}^1$ of *G* with all *wired boundary conditions* in the special case of the critical FK Ising model, that is, when $q=2$ and $p = \sqrt{2}/(1 + \sqrt{2})$. Its dual model is again a critical FK Ising model, now with free boundary conditions on the dual graph $G^\circ$ of *G* which is a (simply connected) subgraph of $\mathbb {L}^\circ$. The *loop representation* is obtained as loops which form boundaries between open cluster and the dual open clusters and is defined as a collection of loops on the corresponding subgraph $G^\spadesuit$ of the modified medial lattice $\mathbb {L}^\spadesuit$. The loop collection satisfies the properties of the following definition. See also Fig. 2 for illustration of the common loop representation shared by the random cluster model and its dual model.

Let’s call a (unordered) collection of loops $\mathcal {L}=(L_j)_{j =1 ,\ldots N}$ on $G^\spadesuit$ a *dense collection of non-intersecting loops* (DCNIL) if

- each $L_j \subset G^\spadesuit$ is a simple loop
- $L_j$ and $L_k$ are vertex-disjoint when $j \ne k$
- for every edge $e \in E^\diamond$ there is a loop $L_j$ that visits *e*. Here we use that $E^\diamond$ is naturally a subset of $E^\spadesuit$.

Let the collection of all the loops in the loop representation be $\Theta =(\theta _j)_{j =1 ,\ldots N}$. Then DCNIL is exactly the support of $\Theta$ and for any DCNIL collection *C* of loops5where *Z* is the partition function that normalizes the probability measure.

We consider two boundaries of the domain, one which is the boundary of the domain and one which shifted by one lattice step from the first one towards the interior of the domain. They are both simple loops on the lattice which satisfy the same parity condition as the loops of the random cluster loop representation (the octagons on both sides have uniform color). More specifically

- $\partial G^\spadesuit$ is the boundary of the domain, in the usual topological sense. Call it the *external boundary*.
- $\partial _1 G^\spadesuit$ is the outermost (simple) loop can be drawn in $G^\spadesuit$. In other words, it is the outermost loop of the empty random cluster configuration with wired boundary conditions. Call it the *internal boundary*. We say that $\partial _1 G^\spadesuit$ touches the boundary everywhere and that a *loop touches the boundary* if if it intersects $\partial _1 G^\spadesuit$. Notice that if a loop and $\partial _1 G^\spadesuit$ intersect then they share an edge (which is an edge shared by two octagons).

Define the collection of *boundary touching loops*, $\Theta _\partial \subset \Theta$, to be simply the set of loops which intersect the internal boundary $\partial _1 G^\spadesuit$.

Recall the Carathéodory convergence from Sect. 1.2.2 a bounded simply connected domain $\Omega$ in the plane. And take a sequence $\delta _n \searrow 0$ as $n \rightarrow \infty$ and a sequence of simply connected graphs $G_{\delta _n}^\bullet \subset \delta _n \mathbb {L}^\bullet$ which approximate $\Omega$ in the sense that, if we denote by $\Omega _{\delta _n}$ the bounded component of $\mathbb {C}{\setminus } \partial G_{\delta _n}^\spadesuit$, then $\Omega _{\delta _n}$ converges in Carathéodory convergence to $\Omega$ (with respect to any interior point of $\Omega$). Fix any $w_0\in \Omega$ and let $\phi _{\delta _n}: \Omega _{\delta _n} \rightarrow \mathbb {D}$ be conformal transformations normalized in the usual way using $w_0$, that is,

$$\begin{aligned} \phi _{\delta _n}(w_0)=0, \quad \phi _{\delta _n}^{\prime }(w_0)> 0 . \end{aligned}$$

Let $\Theta _{\partial ,\delta _n}$ be the collection of boundary touching loops in $\Omega _{\delta _n}$ and set $\tilde{\Theta }_{\partial ,\delta _n} = \phi _{\delta _n} (\Theta _{\partial ,\delta _n})$.

Let us rephrase here the first half of Theorem 1.1.

#### Theorem 1.1 (a)

(Conformal invariance of $\Theta _\partial$). As $n \rightarrow \infty$, $\tilde{\Theta }_{\partial ,\delta _n}$ converges weakly to a random collection $\Theta$ of loops in $\mathbb {D}$. The law of $\Theta$ is independent of $\Omega , \delta _n, \Omega _{\delta _n}$ and $w_0$.

#### Remark 2.2

Notice that the independence of the law of $\Theta$ from $\Omega , \delta _n, \Omega _{\delta _n}$ and $w_0$ implies that $\Theta$ is invariant under all conformal automorphisms (Möbius transformations) of $\mathbb {D}$. The rotational invariance requires a separate argument using the correspondence between exploration tree and the loop collection and the fact that we are free to choose the root for the exploration. See Sect. 6.

### The exploration tree of FK Ising model

Let’s simplify the notation so that we use $\partial \Omega$ and $\partial _1 \Omega$ to denote $\partial G^\spadesuit$ and $\partial _1 G^\spadesuit$. Remember that $\partial \Omega$ and $\partial _1 \Omega$ are simple loops on $\mathbb {L}^\spadesuit _\rightarrow$ and that $\Theta _\partial$ was the set of loops in $\Theta$ that intersected $\partial _1 \Omega$. Next we will explain the construction of the *exploration tree* of $\Theta _\partial$. The branches of the tree will be simple paths from a root edge to a directed edge of $\partial _1 \Omega$. More specifically let $V_{\mathrm{target}}$ be the vertex set of $\partial _1 \Omega$ and for each $v \in V_{\mathrm{target}}$, let $f_v$ be the edge of $\partial _1 \Omega$ arriving to *v*.

We assume that the root vertex $v_{\mathrm{root}}\in V_{\partial }$ of the exploration is fixed. For any $w \in V_{\mathrm{target}}$, we are going to construct a simple path which starts from the inwards pointing edge of $v_{\mathrm{root}}$ and ends on the edge $f_w$ of *w*, denoting this path by $T_w=T_{v_{\mathrm{root}},w}$. We will call the mapping from the loop collections to the trees the “*loops-to-tree map*.”

We describe next the algorithm of Sect. 1.2.1 when the target point is on the boundary. Consider a loop collection $\mathcal {L}=(L_j)_{j \in J}$ where *J* is some finite index set, and assume that $\mathcal {L}$ satisfies the properties of DCNIL. The index $j \in J$ shouldn’t be confused with the concrete sequence $L_1,\ldots ,L_n$ chosen below. Here we consider $L_j$ as a path in $\mathbb {L}^\diamond$ and lift it to $\mathbb {L}^\spadesuit$ when needed.

1. Set $e_0$ to be the inward pointing edge of $\partial _1 \Omega$ at $v_{\mathrm{root}}$ and $f_{\mathrm{end}}=f_w$, that is the edge of $\partial _1 \Omega$ arriving to *w*. Denote the set of edges in $\partial _1 \Omega$ that lie between $f_{\mathrm{end}}$ and $e_0$, including $f_{\mathrm{end}}$, by $F_w$.
2. Set $L_1$ to be the loop going through $e_0$. Find the first edge of $L_1$ after $e_0$ in the orientation (remember that all the loops are oriented in the clockwise direction) of $L_1$ that lies in $F_w$. Call it $f_1$ and the part of $L_1$ between $e_0$ and $f_1$, not including $f_1$, $L_1^\text {T}$. Notice that $L_1$ goes through $f_{\mathrm{end}}$ if and only if $f_1$ is equal to $f_{\mathrm{end}}$. Notice also that if $f_1$ is not $f_{\mathrm{end}}$, then it is the first edge that takes the loop to a component of the domain that is no longer “visible” to $f_{\mathrm{end}}$.
3. Suppose that $f_n$ and $L_1^\text {T}, L_2^\text {T}, \ldots , L_n^\text {T}$ are known. If $f_n$ is equal to $f_{\mathrm{end}}$, stop and return the concatenation of $L_1^\text {T}, L_2^\text {T}, \ldots , L_n^\text {T}$ and $f_n$ as the result $T_w$. Otherwise take the inward pointing $e_n$ edge next to $f_n$ (starting at the endpoint of $f_n$) and the loop $L_{n+1}$ passing through $e_n$. Find the first edge of $L_{n+1}$ after $e_n$ in the orientation of $L_{n+1}$ that lies in $F_w$. Call it $f_{n+1}$ and the part of $L_{n+1}$ between $e_n$ and $f_{n+1}$, not including $f_{n+1}$, $L_{n+1}^\text {T}$. Repeat (3).

The result of the algorithm $T_w$ is called the *exploration process* from $v_{\mathrm{root}}$ to *w*. The collection $\mathcal {T}= (T_w)_{w \in V_{\mathrm{target}}}$ where $V_{\mathrm{target}}=V_\partial$ is called the *exploration tree* of the loop collection $\mathcal {L}$ rooted at $v_{\mathrm{root}}$.

The following result is immediate from the definition of the exploration tree. Two branches coincide until the first time that the branch disconnects the target points by that result, and after that the branches explore disjoint regions, which will later imply independence of this processes for the FK Ising exploration tree.

#### Proposition 2.3

(Target independence of exploration tree). Suppose that $v_{\mathrm{root}}, w, w^{\prime }$ are vertices in $V_\partial$ in counterclockwise order, that can be the same. Let $F_{w,w^{\prime }}$ the edges of $\partial _1 \Omega$ that lie between the outward pointing edges of *w* and $w^{\prime }$ in counterclockwise direction, including the edge at $w^{\prime }$ and but excluding the edge at *w*. Then $T_w$ and $T_{w^{\prime }}$ are equal up until the first edge lying in $F_{w,w^{\prime }}$.

**Fig. 6:**
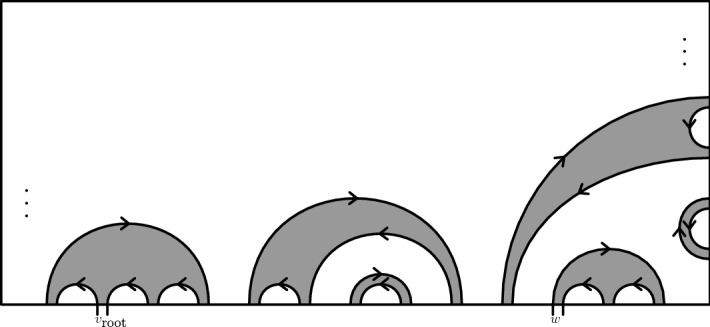
A schematic picture of the boundary touching loops. The interiors of the loops are shaded to make it easier to distinguish the loops and the arrows indicate the clockwise orientation of the loops

Next we will construct the inverse of the loops-to-tree map which we will call a “*tree-to-loops map*.” The structure of the tree $\mathcal {T}$ is the following: the branches of $\mathcal {T}$ are simple and follow the rule of leaving white squares on their right and black on their left. The branching occurs in a subset of vertex set of $\partial _1 \Omega$. There is a one-to-one correspondence between branching points of $\mathcal {T}$ and the boundary touching loops of $\mathcal {L}$: the point on the loop, which lies on the boundary and is the closest one to the root if we move clockwise along the boundary, is a branching point and every branching point has this property for some loop. See also Fig. 6. Suppose that at a branching point *w* the incoming edges are $e_1$ and $e_2$ and the outgoing edges are $f_1$ and $f_2$ and they are in the order $e_1,f_2,e_2,f_1$ clockwise and that the exploration process enters *w* through $e_1$. Then necessarily each of the pairs $e_1, f_1$ and $e_2, f_2$ lie on the same loop of $\mathcal {L}$ and these two loops are different. Also it follows that $f_1$ and $e_2$ are on $\partial _1 \Omega$ while $f_2$ and $e_1$ are not. It follows that the last edge of $T_w$ is $e_2$ and that the part of $T_w$ between $f_2$ and $e_2$ is exactly the loop of $\mathcal {L}$ that touches the boundary at $f_w=e_2$. Doing the same thing for every branching point defines a mapping from a suitable set of trees onto the set of loop collections of boundary touching loops of DCNIL. This mapping inverts the construction of the exploration tree and we summarize it in the following lemma.

#### Lemma 2.4

The mapping from the collection of boundary touching loops $\mathcal {L}_\partial$ to the chordal exploration tree $\mathcal {T}$ is a bijection.

Similar constructions work in the continuous setting. See [23] for the construction of the $\hbox {SLE}(\kappa ,\kappa -6)$ exploration tree and the construction for recovering the loops.

Let us repeat here the second half of Theorem 1.1.

#### Theorem 1.1 (b)

The law of $\Theta$ is given by the image of the $\hbox {SLE}(\kappa ,\kappa -6)$ exploration tree with $\kappa =16/3$ under the above tree-to-loops mapping.

The proof of Theorem 1.1 is given in Sect. 6.

## Tightness of Trees and Loop Collections

In this section, we establish a priori bounds for trees and loop ensembles. The setting is relatively general, although we only apply it here to the FK Ising exploration tree of the boundary touching loops.

### A probability bound on multiple crossings by the tree

An approach to establish compactness properties of sequences of probability measures based on probability bounds of multiple crossings of annuli by random curves was set up in [14] extending the results of [1]. Below we use that type of result for the FK Ising exploration tree. We start from the essential definitions.

For any fixed measurable space $(\mathcal {S},\mathcal {F})$, we call a random variable *X* tight over a collection $\Sigma _0$ of probability measures $\mathbb {P}\in \Sigma _0$ on the space $(\mathcal {S},\mathcal {F})$, if for each $\varepsilon >0$ there exists a constant $M>0$ such that $\mathbb {P}(|X|<M)>1-\varepsilon$ for all $\mathbb {P}$.

A crossing of an annulus $A(z_0,r,R)=\{z \in \mathbb {C}\,:\, r<|z-z_0|<R\}$ is a closed segment of a curve that intersects both connected components of $\mathbb {C}{\setminus } A(z_0,r,R)$ and a minimal crossing doesn’t contain any genuine subcrossings.

Recall the general setup of [14]: we are given a collection $(\phi ,\mathbb {P}) \in \Sigma$ where the conformal map $\phi$ contains also the information about its domain of definition $(\Omega ,v_{\mathrm{root}},w_0)=(\Omega (\phi ),v_{\mathrm{root}}(\phi ),w_0(\phi ))$ through the requirements

6 $$\begin{aligned} \phi ^{-1}(\mathbb {D})=\Omega , \quad \phi (v_{\mathrm{root}})=-1 \quad \text {and} \quad \phi (w_0)=0 \end{aligned}$$

and $\mathbb {P}$ is the probability law of FK Ising model on the discrete domain $\Omega$ and in particular gives the distribution of the FK Ising exploration tree. Given the collection $\Sigma$ of pairs $(\phi ,\mathbb {P})$ we define the collection $\Sigma _\mathbb {D}= \{\phi \mathbb {P}\,:\, (\phi ,\mathbb {P})\in \Sigma \}$ where $\phi \mathbb {P}$ is the pushforward measure defined by $(\phi \mathbb {P}) (E) = \mathbb {P}(\phi ^{-1}(E))$.

#### Theorem 3.1

The following claim holds for the collection of the probability laws of FK Ising exploration trees

- for any $\Delta >0$, there exists $n \in \mathbb {N}$ and $K>0$ such that
  7 $$\begin{aligned} \mathbb {P}( \text {at least } n \text { disjoint segments of } \mathcal {T}\text { cross } A(z_0,r,R)) \le K \left( \frac{r}{R} \right) ^\Delta \end{aligned}$$
  for all $\mathbb {P}\in \Sigma _\mathbb {D}$ and for all $z_0 \in \mathbb {C}$ and $R>r>0$. and there exist positive numbers $\alpha , \alpha ^{\prime }>0$ such that the following claims hold
- if for each $r>0$, $M_r$ is the minimum of all *m* such that each $T\in \mathcal {T}$ can be split into *m* segments of diameter less or equal to *r*, then there exists a random variable $K(\mathcal {T})$ such that *K* is a tight random variable for the family $\Sigma _\mathbb {D}$ and
  $$\begin{aligned} M_r \le K(\mathcal {T}) \, r^{-\frac{1}{\alpha }} \end{aligned}$$
  for all $$r>0$$.
- All branches of $\mathcal {T}$ can be jointly parametrized so that they are all $\alpha ^{\prime }$-Hölder continuous and the Hölder norm can be bounded by a random variable $K^{\prime }(\mathcal {T})$ such that $K^{\prime }$ is a tight random variable for the family $\Sigma _\mathbb {D}$.

Each of the claims have their own applications below although they are closely related, see [1].

#### Proof

We need to verify the first claim and the two other claims follow from it, by results of [1]; more specifically the second claim follows from the reformulated statement presented in the beginning of the proof of Theorem 1.1 of [1] and the third claim follows from Theorem 1.1 of [1]. Notice that we need to verify the inequality (7) for $\Delta = \Delta _{n_i}$ and $n=n_i$ where $n_i$ is an increasing sequence of natural numbers and $\Delta _{n_i}$ is a sequence of positive real numbers tending to infinity, since the left-hand side of the inequality in the first claim is non-increasing in *n*.

Let $A=A(z_0,r,R)$ be annulus, $z_0 \in \mathbb {D}$. Since either $B(z_0,\sqrt{rR}) \subset \mathbb {D}$ or $B(z_0,\sqrt{rR}) \cap \partial \mathbb {D}\ne \emptyset$, it holds that we can choose $r_1= \sqrt{rR}$, $R_1=R$ or $r_1=r$, $R_1= \sqrt{rR}$ such that for $A_1 = A(z_0,r_1,R_1)$, either $A_1 \subset \mathbb {D}$ or $B(z_0,r_1) \cap \partial \mathbb {D}\ne \emptyset$. For that $A_1$ and for $C>1$ big enough, apply the estimate of conformal distortion given by either Lemma A.1 or Lemma A.2, depending on the case, to show that for any $m=1,2,\ldots ,\lfloor \log \left( \frac{R}{r}\right) \rfloor$, there exist $\tilde{A}_m = A(z_m,r_m,2 r_m)$ such that the conformal image of any crossing of $A(z_0,C^{m-1} r, C^m r)$ under $\phi ^{-1}$ is a crossing of $A_m$.

By Lemmas B.1 and B.2 in Appendix B and the results of [7] (in particular, Lemma 5.7) applied to crossings of $\tilde{A}_m$, it follows that for each $\varepsilon >0$ there is *n* such that $\mathbb {P}(\text {at least } n \text { disjoint segments of } \mathcal {T}\text { cross } \tilde{A}_m) < \varepsilon$. Thus (7) holds for *n* and constants $K = \varepsilon ^{-1}$ and $\Delta = \log \frac{1}{\varepsilon }$, Here the constant $\Delta$ tends to $\infty$ as $n\rightarrow \infty$ (i.e. as $\varepsilon$ tends to zero), and the estimates are uniform over all $\mathbb {P}\in \Sigma _\mathbb {D}$ and annuli $A(z_0,r,R)$ with $R>r$. $\quad \square$

### The crossing property of trees

Let $\gamma _k$, $k=0,\ldots ,N-1$, is the collection $\mathcal {T}= ( T_x)$ in a (random) order and suppose that the random curves $\gamma _k$ are each parametrized by [0, 1]. The chosen permutation specifies *the order of exploration* of the curves. More specifically, set $\underline{\gamma }(t) = \gamma _k(t-k)$ when $t \in [k,k+1)$. We call $\underline{\gamma }$ an *explored* collection of branches.

For a given domain $\Omega$ and for a given simple (random) curve $\gamma$ on $\Omega$, we set $\Omega _\tau = \Omega {\setminus } \gamma [0,\tau ]$ for each (random) time $\tau$. Similarly, for a given domain $\Omega$ and for the given finite collection of curves $\gamma _k$ on $\Omega$, we set $\underline{\Omega }_\tau = \Omega {\setminus } \underline{\gamma }[0,\tau ]$ for each (random) time $\tau$.

We call $\Omega _\tau$ or $\underline{\Omega }_\tau$ the domain at time $\tau$.

The following definition generalizes Definition 2.3 from [14]. This definition is needed in order to recognize those crossing events which have low probability.

#### Definition 3.2

For a given domain $(\Omega ,v_{\mathrm{root}})$ and for a given order of exploration (which defines $\underline{\gamma }$) of curves $\gamma _x$, $x \in V_{\mathrm{target}}$, where each curve $\gamma _x$ is contained in $\overline{\Omega }$, starting from $v_{\mathrm{root}}$ and ending at a point *x* in the set $V_{\mathrm{target}}$, define for any annulus $A = A(z_0,r,R)$, for every (random) time $\tau \in [0,N]$ and $x \in V_{\mathrm{target}}$, $A^{\mathrm{u},x}_\tau = \emptyset$ if $\partial B(z_0,r) \cap \partial \underline{\Omega }_\tau = \emptyset$ and

8 $$\begin{aligned} A^{\mathrm{u},x}_\tau = \left\{ z \in \underline{\Omega }_\tau \cap A \,:\, \begin{gathered} \text {the connected component of } z \text { in } \underline{\Omega }_\tau \cap A \\ \text {doesn't disconnect } \underline{\gamma }(\tau ) \text { from } x \text { in } \underline{\Omega }_\tau \end{gathered} \right\} \end{aligned}$$

otherwise. Define also

9 $$\begin{aligned} A^{\mathrm{f},x}_\tau = \left\{ z \in \Omega _\tau \cap A \,:\, \begin{gathered} \text {the connected component of } z \text { in } \underline{\Omega }_\tau \cap A \\ \text {is crossed by any path connecting } \underline{\gamma }(\tau ) \text { to } x \text { in } \underline{\Omega }_\tau \end{gathered} \right\} \end{aligned}$$

and set $A^{\mathrm{u}}_\tau = \bigcap _{x \in V_{\mathrm{target}}}A^{\mathrm{u},x}_\tau$ and $A^{\mathrm{f}}_\tau = \bigcup _{x \in V_{\mathrm{target}}} A^{\mathrm{f},x}_\tau$. We say that $A^{\mathrm{u},x}_\tau$ is *avoidable* for $\gamma _x$ and $A^{\mathrm{u}}_\tau$ is *avoidable for all* (branches). We say that $A^{\mathrm{f},x}_\tau$ is *unavoidable* for $\gamma _x$ and $A^{\mathrm{f}}_\tau$ is *unavoidable for at least one* (branch). Here and in what follows we only consider allowed lattice paths when we talk about connectedness.

#### Remark 3.3

Note that $A^{\mathrm{u}}_\tau \cap A^{\mathrm{f}}_\tau = \emptyset$. This follows from $A^{\mathrm{u},x}_\tau \cap A^{\mathrm{f},x}_\tau = \emptyset$ which holds by definition.

**Fig. 7:**
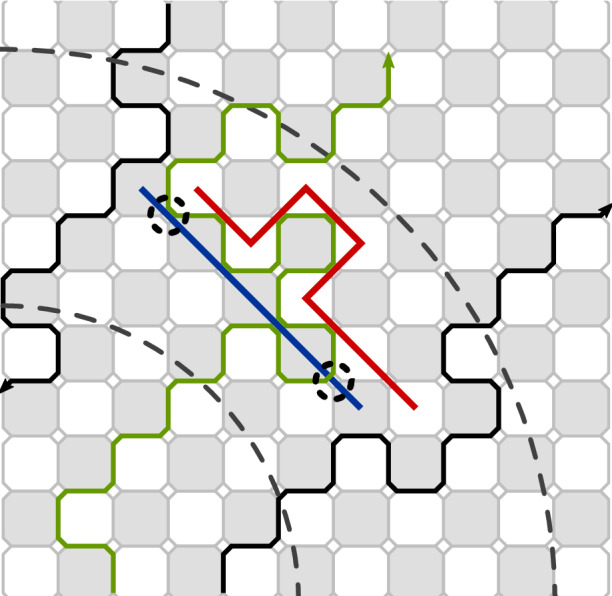
The region between the large dashed circular arcs is a quarter of an annulus. A crossing of an annulus by the exploration path is drawn in green and the boundaries of the domain $\underline{\Omega }_\tau$ in black and they are oriented according to the orientation of the medial lattice. As indicated by the figure if there are transversal open and dual-open paths in the annulus, then the crossing has to go near the left- and right-hand boundaries of the annular sector. Notice that at the branching point on the right-hand side the drawn branch jumps from a loop to another one, whereas on the left-hand side it keeps following the loop explored at that time

Recall that $\partial \Omega$ is the boundary of $\Omega$ and $\partial _1 \Omega$ is the internal boundary of $\Omega$ which is the outermost of all (simple) lattice paths are contained in $\Omega$. A point on $\partial _1 \Omega$ is a *branching point* of the tree $\mathcal {T}$ if it is the last common point of two branches. In that case the edge on the primal lattice passing through the point has to be open in the random cluster configuration. See also Fig. 7.

Next we will write down an estimate in the form of a hypothesis analogous the ones presented in Section 2 of [14]. The estimate is sufficient for the desired compactness properties of the exploration tree. In fact, we will present two equivalent conditions here. As we will later see that conformal invariance will hold for this type of conditions, again analogously to [14]. These conditions will be verified for the FK Ising exploration tree below.

#### Condition G1

Let $\Sigma$ be as above. If there exists $C >1$ such that for any $(\phi ,\mathbb {P}) \in \Sigma$, for any stopping time $0 \le \tau \le N$ and for any annulus $A=A(z_0,r,R)$ where $0 < C \, r \le R$, it holds that

10 $$\begin{aligned} \mathbb {P}\left( \left. \; \begin{gathered} \underline{\gamma }[\tau ,N] \text { makes a crossing of } A \\ \text { which is contained in } \overline{A^u_\tau } \\ \text {or } \\ \text {which is contained in } \overline{A^f_\tau } \text { and the first minimal crossing}\\ \text {doesn't have branching points on both sides} \end{gathered} \;\,\right| \, \mathcal {F}_\tau \right) < \frac{1}{2} . \end{aligned}$$

then the family $\Sigma$ is said to satisfy a *geometric joint unforced–forced crossing bound* Call the event above $E^{\mathrm{u,f}}$.

**Fig. 8:**
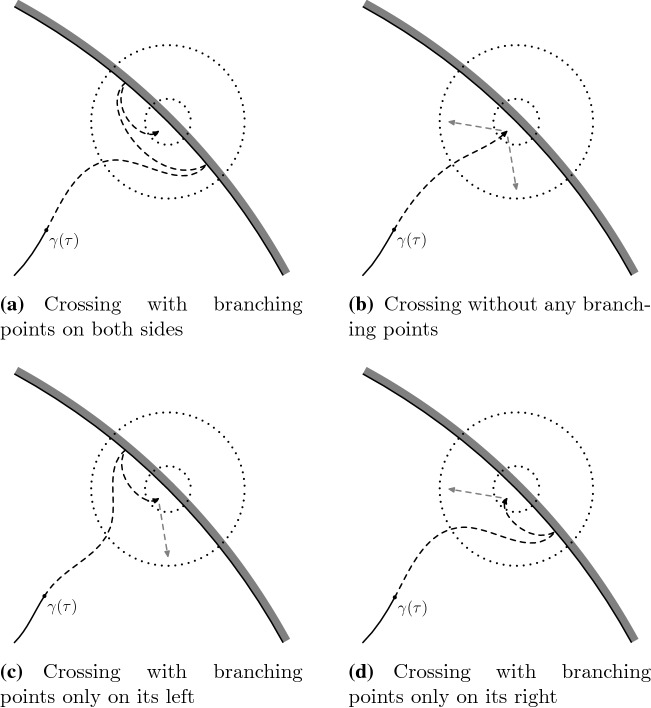
Condition G1 or G2 imply that the crossing events of any of the types illustrated in **b**–**d** has small probability. The longer black arrow is the crossing event considered in (10) and the shorter gray arrows are the crossings of the annulus that are still possible afterwards

See Fig. 8 for more information about different types of branching points.

#### Condition G2

The family $\Sigma$ is said to satisfy a *geometric joint unforced–forced crossing power-law bound* if there exist $K >0$ and $\Delta >0$ such that for any $(\phi ,\mathbb {P}) \in \Sigma$, for any stopping time $0 \le \tau \le N$ and for any annulus $A=A(z_0,r,R)$ where $0 < r \le R$,

11 $$\begin{aligned} \text {LHS} \le K \left( \frac{r}{R} \right) ^\Delta . \end{aligned}$$

Here LHS is the left-hand side of (10).

We want to use Condition G1 or equivalently G2 as a hypothesis for theorems. We start by verifying them for the critical FK Ising model exploration tree.

#### Theorem 3.4

If $\Sigma$ is the collection of pairs $(\phi ,\mathbb {P})$ where $\phi$ satisfies the properties given in (6) and also that its domain of definition $\Omega (\phi )$ is a discrete domain with some lattice mesh, and $\mathbb {P}$ is the law of the critical FK Ising model exploration tree $\mathcal {T}$ on $U(\phi )$, then $\Sigma$ satisfies Conditions G1 and G2.

#### Proof

The theorem can be proved in the same way as the result that a single interface in FK Ising model satisfies a similar condition, which was presented in Section 4.1 of [14]. However, stronger crossing estimates are needed for the exploration tree compared to a single interface. Luckily such estimates were established in Theorem 1.1 of [7]. The full argument goes as follows

**Fig. 9:**
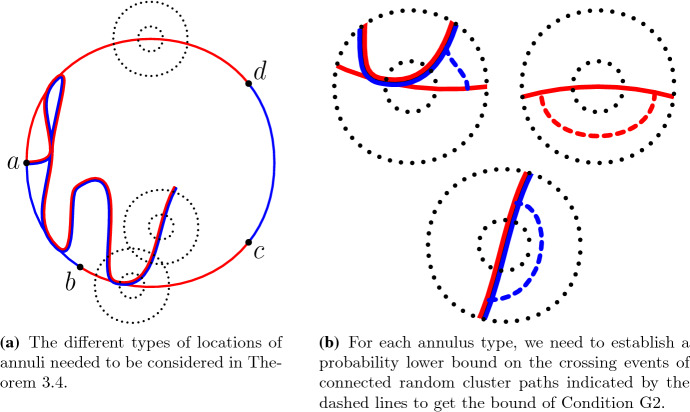
Crossing estimates needed for Theorem 3.4. The setup with four marked points is the same as in Fig. 11 in Sect. 4

- Similarly as in [14], we try to bound uniformly from above the probability of crossings by the interface in an annular sector. This bound can achieved by given a uniform lower bound for open or dual open paths of edges in the random cluster model in the transversal direction. By symmetry we can suppose that we consider open crossings.
- By a similar arguments as in Section 4.1 of [14], we can use FKG inequality to reduce it to a question of open crossing of a topological quadrilateral. See also Figures 12 and 13 in [14]. We can move the wired boundary where the crossing starts and introduce free boundary along the two sides which are parallel to the possible open crossing. However we cannot move the free boundary or replace it by wired boundary if the boundary condition at the endpoint of the possible open crossing is indeed free. Thus we need to consider a general topological quadrilateral and not just regular one (with an archetypical shape), which was enough in [14].
- As indicated by considerations in Fig. 9b, we end up to wired-free-free-free or wired-free-wired-free boundary conditions after the FKG transformation. (That is, we introduce new boundary along the boundaries of the annulus of the opposite “color” as the dashed crossing of the quadrilateral, in the sense that if we consider open crossing the new boundary is free and if the crossing crossing is dual open the new boundary is wired. Then we use the duality as we stated above.)
- Then we use the crossing estimates of [7] to reduce an uniform lower bound. By Theorem 1.1 of [7], the lower bound (as well as the upper bound) are uniform and depend only on the extremal length of the topological quadrilateral.

The upper bound for existence of a curve can improved to the form $C \left( \frac{r}{R} \right) ^\alpha$ using a similar argument as Proposition 2.6 of [14] by considering a disjoint set of concentric annuli where an upper bound of the form $1-\varepsilon$ holds. $\quad \square$

As shown in [14, Proposition 2.6], this type of bounds behave well under conformal maps. We have uniform control on how the constants in Conditions G1 and G2 change if we transform the random objects conformally from one domain to another.

Given a collection $\Sigma$ of pairs $(\phi ,\mathbb {P})$ we define the collection $\Sigma _\mathbb {D}= \{\phi \mathbb {P}\,:\, (\phi ,\mathbb {P})\in \Sigma \}$ where $\phi \mathbb {P}$ is the pushforward measure defined by $(\phi \mathbb {P}) (E) = \mathbb {P}(\phi ^{-1}(E))$.

#### Theorem 3.5

If $\Sigma$ is as in Theorem 3.4, then $\Sigma _\mathbb {D}$ satisfies Conditions G1 and G2.

### Uniform approximation by finite subtrees

#### Topology on trees.

Let $\mathrm {d}_{\mathrm{curve}}$ to be the curve distance (defined to be the infimum over all reparametrizations of the supremum norm of the difference). We define the space of trees as the space of closed subsets of the space of curves and we endow this space with the Hausdorff metric. More explicitly, if $(T_x)_{x \in S}$ and $(\hat{T}_{\hat{x}})_{\hat{x} \in \hat{S}}$ are trees then the distance between them is given by

12 $$\begin{aligned} \mathrm {d}_{\mathrm{tree}}\left( (T_x)_{x \in S}\,,\,(\hat{T}_{\hat{x}})_{\hat{x} \in \hat{S}}\right) = \max \left\{ \sup _x \inf _{\hat{x}} \mathrm {d}_{\mathrm{curve}}\left( T_x,\hat{T}_{\hat{x}}\right) \,,\, \sup _{\hat{x}} \inf _x \mathrm {d}_{\mathrm{curve}}\left( T_x,\hat{T}_{\hat{x}}\right) \right\} . \end{aligned}$$

#### Uniform approximation by finite subtrees.

Let us divide the boundary of the unit disc into a finite number of connected arcs $I_k$, $k=1,\ldots ,N$, which are disjoint, except possibly at their end points. Let $x^\pm _k$ be the end points of $\overline{I_k}$. Suppose that $-1 = \phi (v_{\mathrm{root}})$ is an end point. Naturally it is then an end point of two arcs.

Let $\mathcal {I} = \{ I_k \,:\, k=1,\ldots ,N \}$ and denote the maximum of the diameters of $I_k$ by $m(\mathcal {I})$ and $\{ x^\pm _k \,:\, k=1,\ldots ,N\}$ by $\hat{\mathcal {S}}^\mathbb {D}$. Consider now the random tree $\mathcal {T}_\delta = (T_{v_{\mathrm{root}},x})_x$ whose law is given by $\mathbb {P}_\delta$. The finite subtree $\mathcal {T}_\delta (\mathcal {I})$ is defined by the following steps:

- first take a discrete approximation $\hat{\mathcal {S}}_\delta$ of the set of points $\phi ^{-1}(\hat{\mathcal {S}}^\mathbb {D})$ on the vertex set of $\partial _1 \Omega _\delta$.
- then consider the finite subtree $\hat{\mathcal {T}}_\delta = (T_{v_{\mathrm{root}},x})_{x \in \hat{\mathcal {S}}_\delta }$
- finally set $\mathcal {S}_\delta$ to be the union of $\hat{\mathcal {S}}_\delta$ and all the branching points (in the sense of the definition in Sect. 3.2) of $\hat{\mathcal {T}}_\delta$. Denote $(T_{v_{\mathrm{root}},x})_{x \in \mathcal {S}_\delta }$ by $\mathcal {T}_\delta$. Here $T_{v_{\mathrm{root}},x}$ for a branching point is defined as the subpath of $(T_{v_{\mathrm{root}},x})_{x \in \hat{\mathcal {S}}_\delta }$ that starts from $v_{\mathrm{root}}$ and terminates at *x*.

In other words, we take the subtree corresponding to $\hat{\mathcal {S}}$ and then we augment it by adding all its branching points and the branches ending at those branching points to the tree. We will call below $\mathcal {T}_\delta =\mathcal {T}_\delta (\mathcal {I})$ the *finite subtree* corresponding to $\mathcal {I}$. It is finite in a uniform way over the family of probability laws and domains of definition. Hence the name.

Denote the image of $\mathcal {T}_\delta (\mathcal {I})$ under $\phi$ by $\mathcal {T}^\mathbb {D}_\delta (\mathcal {I})$.

##### Theorem 3.6

For each $$R>0$$

13 $$\begin{aligned} \sup \mathbb {P}_\delta \left[ \,\mathrm {d}_{\mathrm{tree}}\left( \mathcal {T}^\mathbb {D}_\delta (\mathcal {I}) \,,\, \mathcal {T}^\mathbb {D}_\delta \right) \, > R \right] = o(1) \end{aligned}$$

as $m(\mathcal {I}) \rightarrow 0$ where the supremum is taken over $\delta >0$.

##### Remark 3.7

In fact, the supremum in the claim can be taken to be over all shapes $\Omega$, since probability bounds that the proof is based on hold for any shape.

**Fig. 10:**
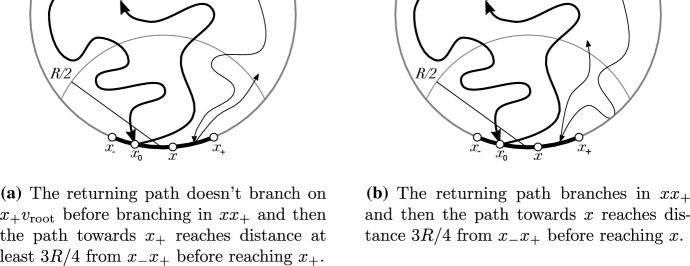
The events which are shown to have low probability in the proof of Theorem 3.6. Two thick arrows are the initial segment of the exploration which visits the boundary first time in $x_- x_+$ at $x_0$ and then exits to distance *R* / 2. The thin arrows indicate the events whose probability is estimated

##### Proof

Let $\varepsilon >0$ and suppose that *K* and $\alpha$ are positive numbers such that $M_r \le K r^{-\frac{1}{\alpha }}$ for all $r>0$ with probability greater than $1-\varepsilon$ for any $\mathbb {P}_\delta$. Such constants exist by Theorem 3.1.

Let $x \in V_{\mathrm{target}}$. Take *k* such that $x \in I_k$. Set $x_\pm = x^\pm _k$. We will show that with high probability $T_{v_{\mathrm{root}},x}$ is close to either $T_{v_{\mathrm{root}},x_-}$, $T_{v_{\mathrm{root}},x_+}$ or some $T_{v_{\mathrm{root}},\tilde{x}}$ where $\tilde{x}$ is a branch point (in the sense of the definition in Sect. 3.2) on the arc $x_- x_+$. We will show that with a high probability this happens for all *x* simultaneously.

Fix $R>0$. We will study under which circumstances

14 $$\begin{aligned} \min \left\{ \mathrm {d}(T_{v_{\mathrm{root}},x}, T_{v_{\mathrm{root}},\tilde{x}}) \,:\, \tilde{x} \in x_- x_+\right\} \end{aligned}$$

is larger than *R* or smaller or equal to *R*. We can assume that $m(\mathcal {I})$ is less than *R* / 10, say. Then the diameter of $x_-x_+$ is at most *R* / 10.

First we notice, that if $x_0$ the first branching point on $I_k$, then the branches to $x,x_-$ and $x_+$ are all the same until they reach $x_0$.

We will consider two complementary cases: either $x_0 \in x_-x$ or $x_0 \in x x_+$.

If $x_0 \in x_-x$, the branches of *x* and $x_+$ continue to be the same after $x_0$ at least for some time. Suppose that $\min \{\mathrm {d}(T_{v_{\mathrm{root}},x}, T_{v_{\mathrm{root}},x_0}),\mathrm {d}(T_{v_{\mathrm{root}},x}, T_{v_{\mathrm{root}},x_+})\}>R$. Then in particular the branch that we follow to continue towards $x_-$ and $x_0$ has to reach distance 3*R* / 4 from $x_-x_+$ without making a branch point in $x v_{\mathrm{root}}$, i.e. a visit to the boundary. It has to contain therefore a subpath which has diameter at least *R* / 2, starts at the boundary and is otherwise disjoint from the boundary. Call the subpaths with this property $\mathcal {J}$. Then by the bound on $M_r$ we get $\#\mathcal {J} \le K (R/2)^{-\frac{1}{\alpha }}$ (see Lemma 2.2 in [1] for the relationship of maximal number of disjoint segments and the minimal number of segments needed to cover a path).

If we happen to reach distance 3*R* / 4 from $x_-x_+$ without making a branch point, then in order $\mathrm {d}(T_{v_{\mathrm{root}},x}, T_{v_{\mathrm{root}},x_+})>R$ to hold the path needs to come close to $x_- x_+$ again and branch on $x x_+$ which it will do surely. But while doing so one of the following has to hold (Fig. 10):

- the returning path doesn’t branch on $x_+ v_{\mathrm{root}}$ before branching in $x x_+$ and then the path towards $x_+$ reaches distance at least 3*R* / 4 from $x_-x_+$ before reaching $x_+$
- or the returning path branches in $x x_+$ and then the path towards *x* reaches distance 3*R* / 4 from $x_-x_+$ before reaching *x*.

By the conditions presented in the previous section the probabilities of both of them can be made less than $\varepsilon$ by choosing length of $x_-x_+$ small.

Now there are $\#\mathcal {J}$ subpaths where this can happen. Hence the total probability is at most

15 $$\begin{aligned} \varepsilon + K (R/2)^{-\frac{1}{\alpha }} \varepsilon = cst.(R) \varepsilon \end{aligned}$$

which can be made arbitrarily small. This argument can be made more formal by introducing stopping times $\tau _n$ such that $\tau _0=0$ and $\tau _n$, $n \ge 1$ is the smallest *t* such that $t> \tau _{n-1}$ and it holds that there exists $s \in [\tau _{n-1},t)$ such that the exploration at time *s* in on the boundary and the exploration on (*s*, *t*) is disjoint from the boundary and has diameter at least *R* / 2. Then if $E_n$ is the event above (described in the two bullets), then the inequality (15) follows simply by using a union bound for $\bigcup _n E_n$.

The other case $x_0 \in x x_+$ is similar. $\quad \square$

### Precompactness of the loops and recovering them from the tree

#### Topology for loops.

Let $\mathbb {T}$ be the unit circle. We consider a loop to be a continuous function on $\mathbb {T}$, considered modulo orientation preserving reparametrizations of $\mathbb {T}$. The metric on loops is given in a similar fashion as for curves. We define

16 $$\begin{aligned} \mathrm {d}_{\mathrm{loop}}(\gamma ,\hat{\gamma }) = \inf \left\{ \left\| f-\hat{f} \right\| _\infty \,:\, f\in \gamma , \; \hat{f}\in \hat{\gamma } \right\} \end{aligned}$$

where we use the notation $f\in \gamma$ to denote that *f* is a parametrization of the (unparametrized) curve $\gamma$. The space of loops endowed with $\mathrm {d}_{\mathrm{loop}}$ is a complete and separable metric space.

A loop collection $\Theta$ is a closed subset of the space of loops. On the space of loop collections we use the Hausdorff distance. Denote that metric by $\mathrm {d}_{\mathrm{LE}}$, where LE stands for *loop ensemble*.

The random loop configuration we are considering is the collection of FK Ising loops which we orient in clockwise direction.

#### Precompactness of the loops.

##### Theorem 3.8

The family of probability laws of $\Theta$ is tight in the metric space of loop collections.

##### Proof

The claim follows directly from Lemma 2.4 and the tightness of the trees. Namely, observe that there exists a constant $\alpha >0$ and a tight random variable $K>0$ such that the minimum number of segments needed to cover any branch in the tree with segments of diameter at most *R* is bounded by $K \,R^{-\alpha }$. By the bijection between loop collections and trees, each loop is a subpath of a branch of the tree and thus we find that the minimum number of segments needed to cover any loop in the loop collection with segments of diameter at most *R* is bounded by $K \,R^{-\alpha }$. $\quad \square$

### Uniform approximation of loops by the finite subtrees

By Lemma 2.4 it is possible to reconstruct the loops from the full tree. And by Theorem 3.6 we can approximate the full tree by finite subtrees. How do we recover loops approximately from the finite subtrees? Can we do it in a uniform manner?

Consider a loop $\theta \in \Theta$. We divide it into *arcs* which are excursions from the boundary to the boundary, otherwise disjoint from the boundary. Since the loop is oriented in the clockwise direction, exactly one of the arcs goes away from $v_{\mathrm{root}}$ in the counterclockwise orientation of the boundary and rest of them go towards $v_{\mathrm{root}}$. The first case is the *top arc*$\gamma ^\text {T}$ of the loop $\gamma$ and the rest of them are the *bottom arcs*. See Fig. 6. The concatenation of the bottom arcs is denoted by $\gamma ^\text {B}$.

Suppose that the loop doesn’t intersect a fixed neigborhood of $v_{\mathrm{root}}^\mathbb {D}$. The diameter of the entire loop is bounded by a uniform constant (depending on the chosen neighborhood) times the diameter of the top arc and vice versa. This means that if the loop has diameter greater than a fixed number, then the top arc is traced by finite subtree for fine enough mesh according to Theorem 3.6.

Also the bottom arcs from right to left are traced until the last branch point to the points defining the finite subtree. Again by Theorem 3.6, the part which remains to be discovered of the loops, has small diameter. Hence if we define approximate loop to have just a linear segment in that place, we see that the loop and the approximation are close in the given metric.

So we define the finite-subtree approximation $\Theta _\delta$ of the random loop collection $\Theta$ to be the collection of all those discovered top arcs concatenated with their discovered bottom arcs and the linear segment needed to close the loop. By Theorem 3.6, we have the following result.

#### Theorem 3.9

For each $$R>0$$

17 $$\begin{aligned} \sup _{ \delta } \mathbb {P}_\delta \left[ \,\mathrm {d}_{ LE }\left( \Theta ^\mathbb {D}_\delta (\mathcal {I}) \,,\, \Theta ^\mathbb {D}\right) \, > R \right] = o(1) \end{aligned}$$

as $$m(\mathcal {I}) \rightarrow 0$$.

### Some a priori properties of the loop ensembles

The following theorem gathers some technical estimates needed below.

#### Theorem 3.10

The family of critical FK Ising loop ensemble measures $(\mathbb {P}_\delta )_{\delta >0}$ satisfy the following properties

- (finite number of big loops) For each $R>0$,
  18 $$\begin{aligned} \sup _{\delta >0} \mathbb {P}_\delta \left( \# \left\{ \gamma \,:\, {{\,\mathrm{diam}\,}}(\gamma ) \ge R \right\} \; \ge N \right) = o(1) \end{aligned}$$
  as $$N \rightarrow \infty $$.
- (small loops and branching points are dense on the boundary) There exists $\Delta >0$ such that for each $x \in \partial \mathbb {D}$ and $R>0$
  19 $$\begin{aligned} \sup _{\delta >0} \mathbb {P}_\delta \left( \sup \left\{ {{\,\mathrm{diam}\,}}(\gamma ) \,:\, \gamma \cap B(x,r) \ne \emptyset \right\} \; \ge R \right) = \mathcal {O}\left( \left( \frac{r}{R} \right) ^\Delta \right) \end{aligned}$$
  as $r \rightarrow 0$. Consequently, for all $c >1$
  20 $$\begin{aligned} \mathbb {P}_\delta \left( \inf \left\{ {{\,\mathrm{diam}\,}}(\gamma ) \,:\, \gamma \cap B(x,c \delta ) \ne \emptyset \right\} \; \ge R \right) = \mathcal {O}\left( \left( \frac{\delta }{R} \right) ^\Delta \right) \end{aligned}$$
  for all $\delta >0$ and $R> c \delta$, and thus for any $\beta \in (0,1)$ and $r>0$ and any partitioning of the boundary of the domain to connected sets $I_j$ of diameter at least *r* it holds that
  21 $$\begin{aligned} \mathbb {P}_\delta \left( \forall j, \; I_j \text { is touched by a loop with diameter at most } \delta ^\beta \right) = 1 - \mathcal {O}\left( \delta ^{\Delta (1-\beta )} \right) \end{aligned}$$
  as $$\delta \rightarrow 0$$.
- (big loops have positive support on the boundary and they touch the boundary infinitely often around the extremal points) For any $R>0$
  22 $$\begin{aligned} \sup _{\delta >0} \mathbb {P}_\delta \left( \exists \gamma \text { s.t. } {{\,\mathrm{diam}\,}}(\gamma ) \ge R \text { and } |x_\text {L}(\gamma ) - x_\text {R}(\gamma )| < r \right) = o(1) \end{aligned}$$
  as $r \rightarrow 0$. Here $x_\text {L}(\gamma )$ and $x_\text {R}(\gamma )$ are the left-most and the right-most points of $\gamma$ along the boundary of the domain (as seen from the root). We call the boundary arc $x_\text {L}(\gamma )x_\text {R}(\gamma )$ the *support of*$\gamma$*on the boundary*. Furthermore, for any constant $0<\eta <1$ there exists a sequence $\delta _0(m)>0$ and constant $\lambda >0$ such that if $x(\gamma )=x_\text {L}(\gamma )$ or $x(\gamma )=x_\text {R}(\gamma )$
  23 $$\begin{aligned} \sup _{\delta \in (0, \delta _0(m))} \mathbb {P}_\delta \left( \begin{gathered} \exists \gamma \text { s.t. } {{\,\mathrm{diam}\,}}(\gamma ) \ge R, |x_\text {L}(\gamma ) - x_\text {R}(\gamma )| \ge r \,\text {and}\; \\ \# \{ n=1,2,\ldots ,m \,:\, \gamma \text { touches boundary} \\ \text {in } A(x(\gamma ),\eta ^n \, r, \eta ^{n-1} \, r) \} \le \lambda m \end{gathered} \right) = o(1) \end{aligned}$$
  as $$m \rightarrow \infty $$.
- (big loops are not pinched)
  24 $$\begin{aligned} \sup _{\delta >0} \mathbb {P}_\delta \left( \begin{gathered} \exists \gamma \text { s.t. } \gamma ^\text {T}= \gamma _1^\text {T}\sqcup \gamma _2^\text {T}, \, \gamma _1^\text {T}\cap \gamma _2^\text {T}= \{x\}\\ {{\,\mathrm{diam}\,}}(\gamma _k^\text {T}) \ge R \text { for } k=1,2 \text { and } {{\,\mathrm{dist}\,}}(x, \partial \mathbb {D}) < r \\ \end{gathered} \right) = o(1) \end{aligned}$$
  and
  25 $$\begin{aligned} \sup _{\delta >0} \mathbb {P}_\delta \left( \begin{gathered} \exists \gamma \text { and } \gamma ^{\prime } \subset \gamma ^\text {B}\text { s.t. } \gamma ^{\prime } = \gamma _1^{\prime } \sqcup \gamma _2^{\prime }, \, \gamma _1^{\prime } \cap \gamma _2^{\prime } = \{x\}\\ \gamma ^{\prime } \cap \partial _1 \Omega =\emptyset , \; {{\,\mathrm{diam}\,}}(\gamma _k^{\prime }) \ge R \text { for } k=1,2 \text { and } {{\,\mathrm{dist}\,}}(x, \partial \mathbb {D}) < r \\ \end{gathered} \right) = o(1) \end{aligned}$$
  as $$r \rightarrow 0$$.
- (big loops are distinct)
  26 $$\begin{aligned} \sup _{\delta >0} \mathbb {P}_\delta \left( \exists \gamma , \tilde{\gamma } \text { s.t. } {{\,\mathrm{diam}\,}}(\gamma ),{{\,\mathrm{diam}\,}}(\tilde{\gamma }) \ge R \text { and } \mathrm {d}_{\mathrm{loop}}(\gamma ,\tilde{\gamma }) < r \right) = o(1) \end{aligned}$$
  as $$r \rightarrow 0$$.

#### Proof

The bound (18) follows from Theorem 3.6. The bound (19) follows directly from the crossing bound of annuli at *x*, and the bound (20) is merely rephrasing the previous bound and specializing to $r= c\delta$. For the bound (21), take $x_j$ to be any point on $I_j$, say, not too close to the endpoints of $I_j$, and use the bound (20) for $x=x_j$, $R=\delta ^\beta$ and sum over *j* to get the an upper bound for an union of events of type in (20); the required bound is obtained as the complement.

The bound (23) is shown to hold by considering the domain $U {\setminus } \underline{\gamma }(\tau )$ where $\tau$ is a stopping time such that the top arc of a big loop $\gamma$ is reaching its endpoint $x(\gamma )$ at time $\tau$, and using the crossing bound of annuli at $x(\gamma )$.

The proofs of (22), (24) and (25) are similar. The exploration process discovers, as seen from the root, the top arc of any loop before the lower arcs. As noted before, the diameter of the top arc is comparable to the diameter of the whole loop. Hence it enough to work with loops that have top-arc diameter more than *R*.

For (22), stop the process when the current arc exits the ball of radius *R* / 4 around the starting point $z_0$ of the arc. The rest of the exploration process makes with high probability a branching point in the annulus $A(z_0,r,R/4)$ before finishing the loop at $z_0$ by the crossing estimate (namely, the estimate (11) used in $A(z_0,r,R/4)$ implies that the path will touch both sides of the of the slit domain boundary inside the annulus before reaching distance *r* from $z_0$). This implies that the event (22) does not occur for that loop. These segments of diameter *R* / 4 in the exploration process are necessarily disjoint (they start on the boundary and remain after that disjoint from the boundary) and hence their number is a tight random variable by Theorem 3.1. The bound (22) follows by a simple union bound.

For (24), stop the process when the diameter of the current arc is at least *R* and the tip lies within distance *r* from the boundary. Let the point closest on the boundary be $z_0$. These arc of diameter *R* are necessarily disjoint: they start from the boundary and remain after that disjoint from the boundary. The rest of the exploration makes with high probability a branching point before it exits the ball of radius *R* centered at $z_0$. The bound (24) follows by a simple union bound.

The proof of (25) is very similar and we omit the details.

For the bound (26), let $\gamma _1$ and $\gamma _2$ be two loops of diameter larger than *R*. Let *S* be the set of points of $\gamma _2$ and let $\gamma _1^\text {T}$ be the top arc of $\gamma _1$ and let $\gamma _1^\text {B}$ be the rest of $\gamma _1$. Without loss of generality, we can assume that $\gamma _1^\text {T}$ separates *S* from $\gamma _1^\text {B}$ in $\mathbb {D}$. Otherwise we can exchange the roles of $\gamma _1$ and $\gamma _2$. Suppose that $\mathrm {d}_{\mathrm{loop}}(\gamma _1,\gamma _2) < r$. Then we claim that the Hausdorff distance of $\gamma _1^\text {T}$ and $\gamma _1^\text {B}$ is less than 2*r*. The distance from any point of $\gamma _1^\text {B}$ to $\gamma _1^\text {T}$ is less than *r*. This follows when we notice that any line segment connecting $\gamma _1^-$ to *S* intersects $\gamma _1^+$. Due to topological reasons, since $\gamma _1$ and $\gamma _2$ are oriented and $\mathrm {d}_{\mathrm{loop}}$ uses this orientation, it also holds that the distance from any point of $\gamma _1^\text {T}$ to $\gamma _1^\text {B}$ is less than 2*r*. Namely, any point in $\gamma _1^\text {T}$ has a point of $\gamma _2$ in its *r* neighborhood, which has distance less than *r* to $\gamma _1^\text {B}$. We can now use the exploration process to explore $\gamma _1^\text {T}$. Take a point $z_0 \in \gamma _1^\text {T}$ that has distance at least $\sqrt{rR}$ to the boundary. Such a point exists by (22) and (25). Now by the crossing property used in $A(z_0,r,\sqrt{rR})$ at the stopping time when the exploration has explored fully $\gamma _1^\text {T}$, with high probability the exploration of $\gamma _1^\text {B}$ doesn’t come close to $z_0$. $\quad \square$

#### Some consequences.

In the discrete setting we are given a tree–loop ensemble pair. The tree and the loop ensemble are in one to one correspondence as explained earlier. Recall that, given the loop ensemble, the tree is recovered by the exploration process which follows the loops in counterclockwise direction and jumps to the next loop at boundary points where the loop being followed turns away from the target point. Recall also that the loops are recovered from the tree by noticing that the leftmost point in the loop corresponds to a branching point of the tree and the rest of the loop is the continuation of the branch to the point just right of that branching point.

Consider any random tree–loop ensemble pair which is a subsequential weak limit of the tree–loop ensemble pairs of the FK Ising model. Since the discrete collections of loops are have finite number of big loops with the uniform bound (18), the loop collection is almost surely at most countable also in the limit (use the Portmanteau theorem for the closed event that there are at most *n* loops of diameter strictly greater than *R*). By the properties of the loop ensemble given in Theorem 3.10, the limiting pair and the process of taking the limit have the following properties

- the loops are distinguishable in the sense that there is no sequence of pairs of distinct loops that would converge to the same loop.
- Each loop consists of a single top arc which is disjoint from the boundary except at the endpoints and a non-empty collection of bottom arcs. In particular, the endpoints of the top arc (the leftmost and rightmost points of the loop) are different.

From these properties we can prove the following result. The second assertion basically means that there is a way to *reconstruct the loops from the tree* also in the limit.

##### Theorem 3.11

Let $(\Theta ^\mathbb {D},\mathcal {T}^\mathbb {D})$ be the almost sure limit of $(\Theta _{\delta _n}^\mathbb {D},\mathcal {T}_{\delta _n}^\mathbb {D})$ as $n \rightarrow \infty$. Write $\Theta ^\mathbb {D}=(\theta _j)_{j \in J}$ and $\Theta _{\delta _n}^\mathbb {D}= (\theta _{n,j})_{j \in J}$ (with possible repetitions) such that almost surely for all $j \in J$, $\theta _{n,j}$ converges to $\theta _j$ as $n \rightarrow \infty$, and then set $x_{n,j}$ to be the target point of the branch of $\mathcal {T}_{\delta _n}^\mathbb {D}$ that corresponds to $\theta _{n,j}$ in the above bijection (described in the beginning of the Sect. 3.6.1). Then

- Almost surely all $x_{n,j}$ converge to some points $x_{j}$ as $n\rightarrow \infty$ and all the branches $T_{x_{n,j}^+}$ converge to some branches denoted by $T_{x_{j}^+}$ as $n\rightarrow \infty$. Furthermore, $x_{j}$ are distinct and they form a dense subset of $\partial \mathbb {D}$ and $\mathcal {T}$ is the closure of $(T_{x_{j}^+})_{j \in J}$.
- On the other hand, $(T_{x_{j}^+})_{j \in J}$ is characterized as being the subset of $\mathcal {T}$ that contains all the branches of $\mathcal {T}$ that have a doublepoint on the boundary. Furthermore, that doublepoint is unique and it is the target point (that is, endpoint) of that branch.
- Any loop $\theta _j$ can be reconstructed from $(T_{x_{j}^+})_{j \in J}$ in the following way: the leftmost point of $\theta _j \cap \partial \mathbb {D}$ (the point closest to the root in the counterclockwise direction) is one of the doublepoints $x^+_j$ and the loop $\theta _j$ is the part between the first and last visit to $x^+_j$ by $T_{x_{j}^+}$.

### Precompactness of branches and description as Loewner evolutions

#### Precompactness of a single branch.

We know now that the sequence of exploration trees is tight in the space of curve collections, which has the topology of Hausdorff distance on the compact sets of the space of curves. This enables us to choose for any subsequence a convergent subsequence. However, it turns out that we need stronger tools to be able to characterize the limit. We will review the results of [14] that we will use.

The hypothesis of [14] is similar to the conditions in Sect. 3.2. Once that hypothesis holds for a sequence of random curves, it is shown in that paper that the sequence is tight in the topology of the space of curves. Furthermore, it is established that such a sequence is also tight in the topology of uniform convergence of driving terms of Loewner evolutions in such a way that mapping between curves and Loewner evolutions is uniform enough so that if a sequence converges in both of the above mentioned topologies the limits have to be the same.

The hypothesis of [14] for the FK Ising branch has been already established since we can see it as a special case of Theorem 3.4.

##### Theorem 3.12

(Kemppainen–Smirnov, [14]). Under certain hypothesis, a sequence probability laws $\mathbb {P}_n$ of random curves of $\mathbb {H}$ has the following properties: for each $\varepsilon >0$, there exists an event *K* such that $\inf _n \mathbb {P}_n(K) \ge 1 - \varepsilon$ and the capacity-parametrized curves in $K \cap \{\gamma \text { simple}\}$ form an equicontinuous family, their driving processes form an equicontinuous family and finally $|\gamma (t)| \rightarrow \infty$ as $t \rightarrow \infty$ uniformly. Moreover the driving processes on the event $K \cap \{\gamma \text { simple}\}$ are $\beta$-Hölder continuous with a bounded Hölder constant for any $\beta \in (0, \frac{1}{2})$.

In addition to [14], see also Section 6.3 in [13] for this type of argument in the case of site percolation.

#### Precompactness of finite subtrees.

For fixed finite number of curves it is straightforward to generalize Theorem 3.12. In fact, the conclusions of Theorem 3.12 hold for any finite subtree that we considered in Sect. 3.5.

In the rest of the paper we use these tools available for us and aim to characterize the scaling limits of finite subtrees of the exploration tree. If we manage to establish the uniqueness of the subsequent scaling limit of those objects, then Theorem 1.1 follows from tightness and from the approximation result, Theorem 3.9.

## Preholomorphic Martingale Observable

### The setup for the observable

**Fig. 11:**
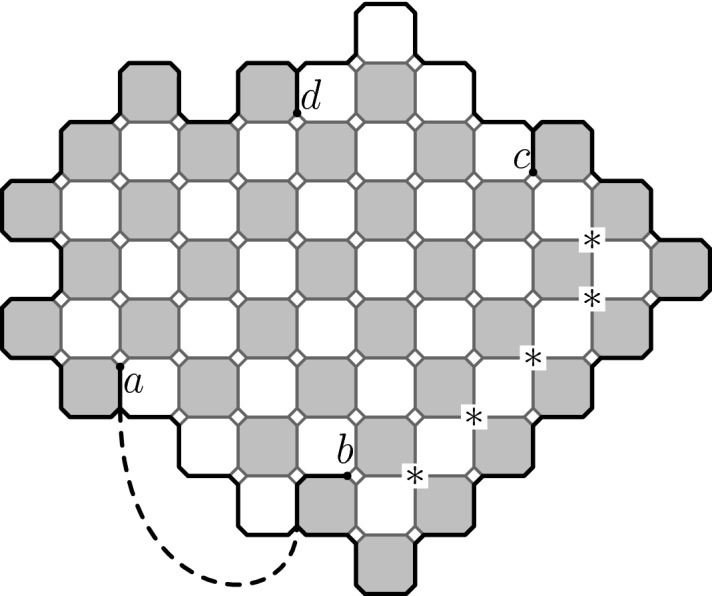
Generalized setting for the chordal exploration tree. We add an external arc from *b* to *a* with the following interpretation. The observable contains an indicator factor for the curve starting from *c* and a complex factor depending on the winding of that curve. If that curve goes to *b* we continue to follow it through the external arc to *a* and from there to *d*. On the other hand, if the curve starting from *c* ends directly to *d*, then the curve connecting *a* to *b* is counted as a loop giving an additional weight $\sqrt{2}$ to the configuration. The arc $bc \subset V_{\partial ,1}$ is marked with $*$’s. Notice that the general domain can have common parts for different boundary arcs longer than one lattice step such as near *b*. In fact, we need to consider domains in this generality, if we wish to explore the interfaces starting at the marked points and the corresponding observables conditioned on the information of the progressing exploration

It is natural to generalize the setup of the previous section to domains of type illustrated in Fig. 11.

Henceforth we consider $G^\spadesuit$ with four special boundary vertices *a*, *b*, *c*, *d*, where the boundary edges (when consider as edges of the directed graph $G^\spadesuit _\rightarrow \subset \mathbb {L}^\spadesuit _\rightarrow$) at *a* and *c* point inwards and the boundary edges at *b* and *d* outwards. Now *a* and *d* play the roles of $v_{\text {root}}$ and *w*, respectively, in the construction of the exploration tree of the previous section. The arcs *ab* and *cd* have white boundary ($\mathbb {L}^\circ$ wired) and *bc* and *da* have black boundary ($\mathbb {L}^\bullet$ wired).

To get a random cluster measure on $G^\spadesuit$ consistent with the all wired boundary conditions and the exploration process in Sect. 2, we have to count the wired arcs *bc* and *da* to be in the same cluster. This corresponds to the external arc configuration where *a* and *b* are connected by an arc and *c* and *d* are connected by an arc. Denote such *external arc pattern*$(a \smile b, c \smile d)$. Later we will denote *internal arc patterns* by $(a \frown b, c \frown d)$ etc.

In fact, we will choose not to draw the external arc $c \smile d$. The reason for this is that it is not used in the definition of the observable and the weights for loop configurations that we get either with or without $c \smile d$ are all proportional by the same $\sqrt{2}$ factor. Thus it doesn’t change the probability distribution.

The configuration on $G^\spadesuit$ is $(\gamma _1,\gamma _2,l_i \,:\, i=1,2,\ldots N_{\mathrm{loops}})$, where $\gamma _1$ and $\gamma _2$ are the paths starting from *a* and *c*, respectively. Define

27 $$\begin{aligned} \hat{\gamma } = {\left\{ \begin{array}{ll} \gamma _2 &{} \text {if } \gamma _2 \text { exits through } d \\ \gamma _2 \sqcup \alpha \sqcup \gamma _1 &{} \text {if } \gamma _2 \text { exits through } b \end{array}\right. } \end{aligned}$$

where $\alpha$ is the planar curve that realizes the exterior arch $a \smile b$ as in Fig. 11 and $\sqcup$ denotes the concatenation of paths. The first case in (27) is the internal arc pattern $(a \frown b, c \frown d)$ and the second is $(a \frown d, c \frown b)$.

For a sequence of domains $G_\delta ^\spadesuit \subset \delta \mathbb {L}^\spadesuit$ with $a_\delta ,b_\delta ,c_\delta ,d_\delta$, define the observable as

28 $$\begin{aligned} f_\delta ( e ) = \theta _\delta \mathbb {E}_\delta ( \mathbbm {1}_{e \in \hat{\gamma }} e^{- i \frac{1}{2} W(d_\delta ,e)} ) \end{aligned}$$

for any $e \in E(G^\diamond _\delta ) \subset E(G^\spadesuit _\delta )$. Here $W(d_\delta ,e)$ is the winding from boundary edge of $d_\delta$ to *e* along the reversal of $\hat{\gamma }$ and $\theta _\delta$ (satisfying $|\theta _\delta |=1$) is a constant, whose value we specify later. Notice that $f_\delta$ doesn’t depend on the choice of $\alpha$ since the winding is well-defined modulo $4 \pi$.

### Preholomorphicity of the observable

**Fig. 12:**
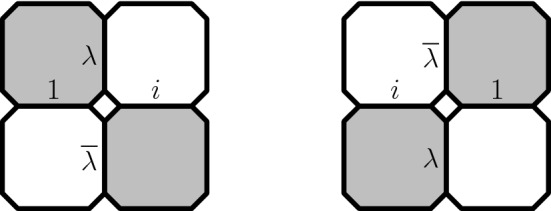
The associated lines $\mathbb {R}, i \mathbb {R}, \lambda \mathbb {R}, \overline{\lambda } \mathbb {R}$ around the two different types of vertices of $\mathbb {L}^\diamond$. Note that for a directed edge $e \in \mathbb {L}^\diamond _\rightarrow$ the line in the complex plane is $\sqrt{\overline{e}} \, \mathbb {R}$ where *e* is interpreted as complex unit vector in its direction

Let $\lambda =e^{-i\frac{\pi }{4}}$. Associate to each edge *e* of the modified medial lattice one of the following four lines through the origin $\mathbb {R}, i \mathbb {R}, \lambda \mathbb {R}, \overline{\lambda } \mathbb {R}$ as in Fig. 12. Denote this line by *l*(*e*).

**Spin preholomorphicity** Choose the constant $\theta$ in the definition of $f_\delta$ so that the value of $f_\delta$ at the edge *e* belongs to the line *l*(*e*). Then $\theta \in \{\pm 1, \pm i, \pm \lambda , \pm \overline{\lambda } \}$. The ± sign mostly doesn’t play any role, but in some situations it should be chosen consistently. The observable $f_\delta$ satisfies the relation

29 $$\begin{aligned} f_\delta (e_W) + f_\delta (e_E)=f_\delta (e_S) + f_\delta (e_N) \end{aligned}$$

for every vertex *v* of the medial graph, whose four neighboring edges in counterclockwise order are called $e_N,e_W,e_S,e_E$. The relation is verified using the same involution among the loop configurations as in [26].

Therefore, using (29), we can define $f_\delta (v) \mathrel {\mathop :}= f_\delta (e_W) + f_\delta (e_E)=f_\delta (e_S) + f_\delta (e_N)$ and it satisfies for any neighboring vertices *v*, *w* the identity

30 $$\begin{aligned} {{\,\mathrm{Proj}\,}}_e (f_\delta (v)) = {{\,\mathrm{Proj}\,}}_e (f_\delta (w)) \end{aligned}$$

where *e* is the edge between *v* and *w* and ${{\,\mathrm{Proj}\,}}_e$ is the orthogonal projection to the line *l*(*e*). That is, if $l(e)=\eta _e \mathbb {R}$ where $\eta _e$ is a complex number with unit modulus, then

31 $$\begin{aligned} {{\,\mathrm{Proj}\,}}_e (z) = {{\,\mathrm{Re}\,}}[z \,\eta _e^*] \,\eta _e = \frac{z + z^* \eta _e^2}{2} . \end{aligned}$$

Since $f_\delta$ on $V(G^\diamond _\delta )$ satisfies the relation (30), we call it *spin-preholomorphic* (or *strongly preholomorphic*). Spin-preholomorphic functions satisfy a discrete version of the Cauchy–Riemann equations [26].

### Martingale property of the observable

Let $\gamma = T_{a,d}$ where $T_{a,d}$ is the branch of the exploration tree constructed in Sect. 2.3. Let’s parametrize $\gamma$ by lattice steps so that at integer values of the time, $\gamma$ is at the head of an oriented edge (in $\mathbb {L}^\spadesuit _\rightarrow$) between black and white octagon and at half-integer values of the time, it is at the tail of such an edge. Note that step between the times $t=k-1/2$ and $t=k$, $k =1,2,\ldots$, is deterministic given the information up to time $t = k-1/2$. Hence between two consecutive integer times, at most one bit of information is generated: namely, whether the curve turn left or right in an “intersection”. Even this choice might be predetermined, if we are visiting a boundary vertex or a vertex visited already before.

Denote the loop configuration by $\omega$, in the four marked point setting it consist of two arcs and a number of loops. We note that the pair $(\gamma [0,t],\omega )$ can be sampled in two different ways (which are basically describing the conditional distributions of one given the other):

1. In the first option, we sample first $\omega$ and then $\gamma [0,t]$ is a deterministic function of $\omega$ as explained above.
2. In the second option, we sample first $\gamma [0,t]$ (or rather we keep the sample of $\gamma [0,t]$ of the previous construction) and then we (re-) sample $\omega ^{\prime }$ (We can rename $\omega ^{\prime }$ in the end as $\omega$. The prime symbol is only used so that there is no confusion with $\omega$ defined above.) in two steps: we sample $\omega ''$ in the complement of $\gamma [0,t]$ using the boundary condition given by $\gamma [0,t]$ and then for each visit of $\gamma [0,t]$ to the arc $bc \subset V_{\partial ,1}$ we flip an independent coin $\zeta _v \in \{0,1\}$, $v \in bc$, such that
  32 $$\begin{aligned} \mathbb {P}(\zeta _v=0) = \frac{1}{1 + \sqrt{2}} , \quad \mathbb {P}(\zeta _v=1) = \frac{\sqrt{2}}{1 + \sqrt{2}} \end{aligned}$$
  and then we open for each visited $v \in bc$ such that $\zeta _v=1$ the edge $e_v \in E(\mathbb {L}^\bullet )$ in $\omega ^{\prime \prime } \cup \gamma [0,t]$ and call the resulting configuration $\omega ^{\prime }$. Notice that the numbers on the right-hand sides in (32) are $1-p_c$ and $p_c$, repectively. The number $1-p_c$ is the weight of the configuration where the edge at *v* is closed relative to the configuration where the edge is open.

See Table 1 for details about the equivalence of these two constructions of $(\gamma [0,t],\omega )$. The configurations are grouped in Table 1 in pairs so that the configurations only differ by the state of the edge at *v*. By construction, the right column occurs for $\gamma$ surely, since the left column leads to branching at *v*. The relative weights of the $\mathbb {L}^\bullet$-edge at *v* being open or closed are independent of are calculated in the table giving (32); namely, the relative weight of the pair of the configuration is a constant. Notice also that *bc* mattered only here since when the branch hits *ab* or *da* (or even *cd*, if we continue to process all the way up to *d*) it continuous to follow the same “loop”. Thus the loop-to-loop jump can only occur on *bc*.

**Table 1:**
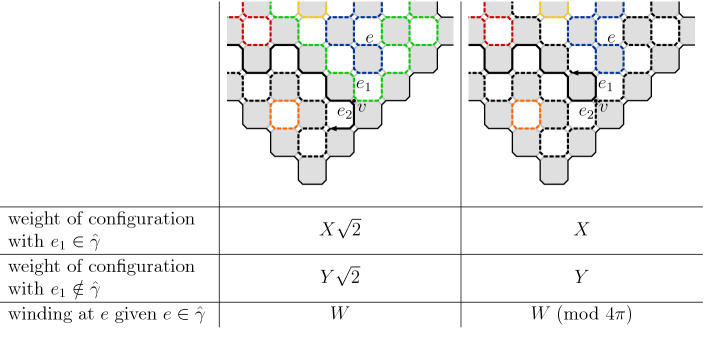
The effect of a jump at a vertex *v* to the weight of a loop configuration and the winding of $\hat{\gamma }$

The involution where the state of the random cluster configuration is changed at *v* and which is illustrated in this pair of figures, preserves the winding factor in the observable and the relative weight of the configuration. All the edges mentioned are in $E(G^\diamond _\delta )$, that is, they are edges between two octagons. The edges $e_1$ and $e_2$ are edges starting at a boundary vertex *v* (a vertex along the internal boundary). The edge *e* is any fixed edge on the graph, and it is the point at which we consider the value of the observable

For a non-negative integer *t*, let $G_t^\spadesuit$ be the slit graph where we have removed $\gamma [0,t]$ and let $a_t$ be the edge $\gamma ([t-1/2,t])$.

Next we decorate the boundary arc $bc \subset V_{\partial ,1} (G^\diamond )$ with i.i.d. random variables $(\xi _v)$, $\xi _v \in \{-1,1\}$, *v* a vertex on *bc*, such that

33 $$\begin{aligned} \mathbb {P}( \xi _v = -1 )=1 - \frac{1}{\sqrt{2}}, \quad \mathbb {P}( \xi _v = 1 )= \frac{1}{\sqrt{2}} . \end{aligned}$$

Let $(\mathcal {F}_t)_{t \in \mathbb {R}_+}$ be the filtration such that the $\sigma$-algebra $\mathcal {F}_t$ is generated by $\gamma [0,t]$ and $\xi _v$ for any $v \in \gamma [0,t-1] \cap bc$.

Notice that we can couple $\zeta _v$ and $\xi _v$ in such a way that

34 $$\begin{aligned} \mathbb {P}( \xi _v = 1 \,|\, \zeta _v=0)=1, \quad \mathbb {P}( \xi _v = 1 \,|\, \zeta _v=1)=\mathbb {P}( \xi _v = -1 \,|\, \zeta _v=1)=\frac{1}{2} . \end{aligned}$$

We interpret $\zeta _v$ so that $\zeta _v = 1$ if and only if we jump at *v* from one boundary touching loop to the neighboring one, and $\xi _v$ so that $\xi _v=1$ when there is no jump and $\xi _v$ is a fair coin flip when there is a jump.

Set for any integer *t*

35 $$\begin{aligned} M_t^+&= \mathbb {P}^{G^\spadesuit _t,a_t,b_t,c,d}( \gamma _1 \subset \hat{\gamma }) \end{aligned}$$

36 $$\begin{aligned} M_t&= \left( \prod _{v \in \gamma [0,t-1] \cap bc} \xi _v \right) M_t^+ \end{aligned}$$

where we can either take $b_t=b$ or $b_t$ is the rightmost point visible from *cd*. The rightmost visible point means that we move $b_t$ on all branching events to the place where we cut the next loop open, and to the edge which points towards that vertex, to be more specific. That is, $M_t^+$ is the probability that *a* is connected to *d* by the interface (internal arc) in the slit domain and by the proof of the next result, it is interpreted as a conditional probability in the original domain. Lemmas 4.1 and 4.2 below are very central for the proof of the main theorem, namely, the law of the exploration process is determined from the martingale property of these quantities.

#### Lemma 4.1

$$(M_t)$$ is $$(P,\mathcal {F}_t)$$ martingale.

#### Proof

First notice that if $\gamma (t) \notin bc$, then $\mathbb {E}[ M_{t+1}^+ \,|\, \mathcal {F}_t] = M_t^+$ by the Markov property of random cluster model and hence

37 $$\begin{aligned} \mathbb {E}[ M_{t+1} \,|\, \mathcal {F}_t] = \left( \prod _{v \in \gamma [0,t-1] \cap bc} \xi _v \right) \mathbb {E}[ M_{t+1}^+ \,|\, \mathcal {F}_t] = M_t . \end{aligned}$$

On the other hand, if $\gamma (t) \in bc$, then $M_{t+1}^+ = (1+\sqrt{2}) \, M_t^+$ by (32), in other words, $M_{t+1}^+$ would hit 0 if $\gamma$ continued to follow a loop which turns away from *d*, and thus the other possible value of $M_{t+1}^+$ (when $\gamma$ turns towards *d*) has to be by the Markov property of random cluster model and consequent martingale property, equal to $(1+\sqrt{2}) \, M_t^+$ which is one over the probability of that event times the value of $M_t^+$. Hence

38 $$\begin{aligned} \mathbb {E}[ M_{t+1} \,|\, \mathcal {F}_t] = \left( \prod _{v \in \gamma [0,t-1] \cap bc} \xi _v \right) M_t^+ \, \underbrace{(1+\sqrt{2}) \, \mathbb {E}[ \xi _{\gamma (t)} \,|\, \mathcal {F}_t]}_{=1} = M_t . \end{aligned}$$

Therefore $M_t$ is a martingale. $\quad \square$

Define for fixed edge *e*

39 $$\begin{aligned} N_t = \theta _\delta \, \mathbb {E}^{G^{\spadesuit }_t,a_t,b_t,c,d} (\mathbbm {1}_{e \in \hat{\gamma }} e^{-i \frac{1}{2} W(d,e)} ) \end{aligned}$$

where $\theta _\delta$ is the constant with unit modulus chosen in Sect. 4.2. In words, the value of the process $(N_t)$ at time *t* is the value at the given edge *e* of the observable on the slit domain at time *t*. For the next property $\theta _\delta$ needs to be chosen consistently. This can be done for example by requiring that the sign of the observable is always the same at *d*.

#### Lemma 4.2

$$(N_t)$$ is $$(P,\mathcal {F}_t)$$ martingale.

#### Proof

If $\gamma (t) \notin bc$, then clearly $\mathbb {E}[ N_{t+1} | \mathcal {F}_t] = N_t$.

If $\gamma (t) \in bc$, then $N_{t+1} = N_t$, since by the observations in Table 1 the random variables $\mathbbm {1}_{e \in \hat{\gamma }}$ and $e^{-i \frac{1}{2} W(d,e)}$ are independent from the state of the edge at $\gamma (t)$ conditionally on the history up to time *t*. Therefore $N_t$ is a martingale. $\quad \square$

### Convergence of the observables

In this subsection we first work on the scaling limit of the *4-point observable*. In that approach we keep the points $c_\delta$ and $d_\delta$ at a macroscopically positive distance. That could be considered as a motivation for the so called *3-point* or *fused observable* where we take the same limit, but keeping $c_\delta$ and $d_\delta$ at a microscopically bounded distance.

**Fig. 13:**
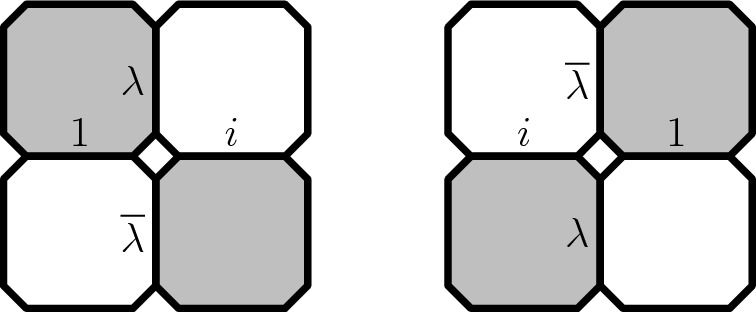
The setup for the random cluster measure, for the curves and loops and the boundary conditions satisfied by the (discrete) harmonic functions

#### Convergence of the 4-point observable.

It is straightforward to apply the reasoning of [26] to this case. We only summarize the method here without any proof. See also the lecture notes [11].

The observable $f_\delta$ is given on the medial lattice. It is preholomorphic on the vertices of the lattice and it has well-defined projections to the complex lines *l*(*e*) defined on the edges of the lattice. Define a function $H_\delta$ on the square lattice by setting $H_\delta (B_0)=1$ where $B_0$ is the black square next to *d* and then extending to other squares by

40 $$\begin{aligned} H_\delta (B) - H_\delta (W) = |f_\delta (e)|^2 \end{aligned}$$

where *B* and *W* are any pair of neighboring black and white square and *e* is the edge of the medial lattice between them. Now $H_\delta$ is well-defined since by the properties of $f_\delta$ the sum of differences of $H_\delta$ along a closed loop is zero. The boundary values $H_\delta$ are the following, see also Fig. 13:

- $H_\delta$ is equal to 0 on the arc *cd*.
- $H_\delta$ is equal to 1 on the arcs *da* and *bc*.
- $H_\delta$ is equal to $1-\beta _\delta$ on the arc *ab*.

The relation between $f_\delta$ and $H_\delta$ becomes more clear after a small calculation. Namely, by this calculation for neighboring black squares $B,B^{\prime }$ with a vertex $v \in L_\diamond$

41 $$\begin{aligned} H_\delta (B^{\prime }) - H_\delta (B) = \frac{1}{2} {{\,\mathrm{Im}\,}}\left( f_\delta (v)^2 \,\frac{B^{\prime }-B}{\delta } \right) . \end{aligned}$$

Notice that the complex number $(B^{\prime }-B)/\delta$ has modulus $\sqrt{2}$. The natural interpretation is that $H_\delta (B)$ is the imaginary part of the discrete integral (counted in lattice-step units) of $\frac{1}{2}f_\delta ^2$ from $B_0$ to *B* along any connected path of black squares. This means that

42 $$\begin{aligned} H_\delta (B^{\prime }) = H_\delta (B) + {{\,\mathrm{Im}\,}}\int _B^{B^{\prime }} \left( \frac{1}{\sqrt{2\delta }} f_\delta \right) ^2 \end{aligned}$$

where the integral sign denotes the discrete integral is over any lattice path connecting *B* to $B^{\prime }$ and is defined as the sum over the edges of the path, of the integrand evaluated at the mid point of the edge times the complex number which is the difference of the head and the tail of the edge.

Denote the restriction of $H_\delta$ to black and white squares by $H_\delta ^\bullet$ and $H_\delta ^\circ$, respectively. Again by the properties of $f_\delta$, $H_\delta ^\bullet$ is subharmonic and $H_\delta ^\circ$ is superharmonic, see [26, Lemma 3.8]. Let $\tilde{H}_\delta ^\bullet$ be the preharmonic function on the black squares with the same boundary values as $H_\delta ^\bullet$ and similarly $\tilde{H}_\delta ^\circ$ be the preharmonic extension of the boundary values of $H_\delta ^\circ$. Also extend all these function to be continuous functions, say, by using the bilinear extension which in takes the form

$$\begin{aligned} h(x,y)= a_1 +a_2 x + a_3 y + a_4 x y \end{aligned}$$

in each square and matches with the values given at the corners of the square. Then at each interior point

43 $$\begin{aligned} \tilde{H}_\delta ^\circ (z) \le H_\delta ^\circ (z) \le H_\delta ^\bullet (z) \le \tilde{H}_\delta ^\bullet (z) . \end{aligned}$$

Next apply standard difference estimates to show that the preharmonic functions $\tilde{H}_\delta$ and $\tilde{H}_\delta ^\bullet$ have convergent subsequences and also using crossing estimates show that their boundary values approach to each other. Since $0 \le \beta _\delta \le 1$, by taking a subsequence we assume that $\beta _\delta$ converges to a number $\beta$ and $H_\delta$ converges to a harmonic function on $\Omega$ with the boundary values $H=0$ on *cd*, $H=1$ on *bc* and *da* and $H=1-\beta$ on *ab*.

As explained in [26, Section 5] we can extend the convergence of $H_\delta$ to the convergence of $f_\delta$ and hence along the same subsequence as $H_\delta$ converges to *H* also $\frac{1}{\sqrt{2 \delta }} f_\delta$ converges to *f* defined by

44 $$\begin{aligned} f(z) = \sqrt{\phi ^{\prime }(z)} \end{aligned}$$

where $\phi$ is any holomorphic function with ${{\,\mathrm{Im}\,}}\phi = H$.

In fact, the value of $\beta$ is determined uniquely and it depends only on the conformal type of the domain. We’ll give the argument in Sect. 4.5 for completeness following the lines of Sect. 4.4.2. Suppose for now that it is the case. Then it follows that the whole sequence $\frac{1}{\sqrt{2 \delta }} f_\delta$ converges.

#### A proof for the fused case.

Consider now the same setup, but when *c* and *d* are close to each other, say, at most at bounded lattice distance from each other. Denote by $z_0$ the point in the continuum that *c* and *d* are approximating. We will deal with the case of flat boundary near $z_0$.

We will change the definition of *H* to that of $1-H$, it means that on the arc *ab*, $H \equiv \beta$, on the arcs *bc* and *da*, $H \equiv 0$ and on the arc *cd*, $H \equiv 1$. Then *H* is superharmonic when restricted to $\mathbb {L}^\bullet$ and subharmonic when restricted to $\mathbb {L}^\circ$ and the inequalities in (43) are reversed.

We expect that in the fused (3-point) limit, $\beta _\delta$ goes to zero, which we have to compensate by renormalizing the observable. In effect, the value of *H* on *cd* will go to infinity and we expect to get a Poisson kernel type singularity. Hence we say that there is a singularity at $z_0$ (or at *c* or *d*).

We will make the following definitions:

- The discrete half-plane $\mathbb {H}_\delta ^{(z_0, \theta )}$ is a discrete approximation of $\mathbb {H}^{(z_0, \theta )} \mathrel {\mathop :}=z_0 + e^{i \theta } \mathbb {H}$ where $\mathbb {H}= \{ z \in \mathbb {C}\,:\, {{\,\mathrm{Im}\,}}[z]>0\}$. Suppose that the boundary lies between two parallel lines that are at a distance which remains bounded (in lattice steps) as $\delta \rightarrow 0$. Assume also that the projection of a parametrization of the boundary on one of the lines is a monotone function, at least in sufficiently large neighborhood of $z_0$. This ensures that there are no long fjords near $z_0$.
- $H^{+}_\delta$, $f^+_\delta$ are the half plane functions on $\mathbb {H}_\delta ^{(z_0, \theta )}$ with the “singularity” at $z_0$. That is, $f^+_\delta$ is the unique, up to ± sign, bounded preholomorphic function on $\mathbb {H}_\delta ^{(z_0, \theta )}$ whose boundary values are (i) $\pm 1$ times *l*(*c*) (see Sect. 4.2) on the edge at *c*, (ii) that value transported to *d* by a lattice path connecting *c* to *d* in $\mathbb {H}_\delta ^{(z_0, \theta )}$ on the edge at *d* and (iii) otherwise satisfy the boundary condition that $f^+_\delta$ is parallel to $\frac{1}{\sqrt{\tau (z)}}$ where $\tau (z)$ is the unit tangent at the boundary point *z* (e.g. if the incoming edge is pointing to the direction of the unit complex number $e_1$ and the outgoing edge is pointing to the direction of the unit complex number $e_2$, then the uni tangent is $(e_1 + e_2)/\sqrt{2}$). Such $f^+_\delta$ can be defined also as the usual FK Ising observable in $\mathbb {H}_\delta ^{(z_0, \theta )}$. The function $H^{+}_\delta$ is defined similarly as above by summing $\pm |f^+_\delta (e)|^2$ along lattice paths, and its boundary values are 1 on *cd* and 0 elsewhere.
- Suppose that $\Omega _\delta$ is a discrete domain and $z_0$ is its boundary point. Assume that the boundary in a *r* neighborhood of $z_0$ is flat in the same uniform manner in $\delta$ as in the definition of the half plane $\mathbb {H}_\delta ^{(z_0, \theta )}$.
- $H_\delta$, $f_\delta$ are the functions on $\Omega _\delta$ with the “singularity” at $z_0$.

The next lemma gives the convergence of the observable in a half-plane. We will compare the other observables to this one.

##### Lemma 4.3

Let $w_\delta$ be the discrete approximation of the point $z_0 + i\,e^{i \theta }$. Let $L_\delta = H^+_\delta (w_\delta )$ and $\hat{H}^+_\delta = \frac{1}{L_\delta } H^+_\delta$. The following statements hold:

(i) As $\delta \rightarrow 0$ the sequence $\hat{H}^+_\delta$ converges uniformly on compact sets to ${{\,\mathrm{Im}\,}}( -\frac{e^{i \theta }}{z-z_0})$.
(ii) For each sequence $\delta _n \searrow 0$ as $n \rightarrow \infty$, there exists a subsequence $\delta _{n_k}$ and a constant $c^+ \in \mathbb {R}_{>0}$ such that as $k \rightarrow \infty$ the sequence $L_{\delta _{n_k}}/\delta _{n_k}$ converges to $c^+$ and the sequence $\delta _{n_k}^{-1} H^+_{\delta _{n_k}}$ converges uniformly on compact sets to $c^+ {{\,\mathrm{Im}\,}}( -\frac{e^{i \theta }}{z-z_0})$.

##### Proof

Let’s first prove (i) and then use that result to prove (ii).

We need here that $\hat{H}^+_\delta (w_\delta )=1$ and $\hat{H}^+_\delta \ge 0$. Harnack’s inequality and Harnack boundary principle [10] imply that for any $r>0$, $\hat{H}^+_\delta \le C$ in $\mathbb {H}_\delta ^{(z_0, \theta )} {\setminus } B(z_0,r)$.

Fix a compact subset of $\mathbb {H}^{(z_0,\theta )}$ with non-empty interior. From boundedness of $\hat{H}^+_\delta$ in the whole domain it follows that the corresponding *f* function $\frac{1}{\sqrt{\delta }} \hat{f}^+_\delta$ remains bounded on that compact set, see [11, Section 5.1.3]. The boundary the values of $\hat{H}^+_\delta$ on $\mathbb {L}^\bullet$ and $\mathbb {L}^\circ$ are close and hence the harmonic extensions of the two functions to the interior are close. Hence (by a standard argument) along a subsequence $\hat{H}^+_\delta$ converges to a function on that compact set and the limit is harmonic in the interior points. It follows that $\frac{1}{\sqrt{\delta }} \hat{f}^+_\delta$ converges uniformly on compact subset along that subsequence similarly as in Sect. 4.4.1.

By taking an increasing sequence of compact sets we see that the convergence takes place in the whole half-plane for a subsequence. The limit has to be the Poisson kernel of the half-plane normalized to have value 1 at the point $z_0 + i\,e^{i \theta }$, because the limit is harmonic and $\hat{H}^+_\delta$ is non-negative, has zero boundary values away from $z_0$ and satisfies the normalization $w_\delta$. This claim can be proven using integration with respect to the Poisson kernel of the upper half-plane (shifted by small $\epsilon >0$ towards the interior of the domain). Since the limit is the same for all subsequences, the whole sequence converges.

For (ii) we use the first claim, (i), and the fact that $H^+_\delta = L_\delta \, \hat{H}^+_\delta$. Notice that the harmonic upper and lower bound for the restrictions of $H^+_\delta$ to $\mathbb {L}^\circ$ and $\mathbb {L}^\bullet$ can be bounded from above and from below, respectively, by a quantity of the form $const. \delta$ just by considering the probabilities of simple random walks to exit the domain through *cd* on these two lattices. Notice that we use here the fact that the boundary is flat around $z_0$ (though such bounds hold also true, when the boundary is smooth and the approximating discrete boundary is well chosen, see also Remark 4.6). The best constants that we get might have a gap in between. Nevertheless, there exist constants $C_1$ and $C_2$ such that $0< C_1< \delta ^{-1} L_\delta< C_2 < \infty$ for small enough $\delta$.

Thus we can take a subsequence $\delta _n$ so that $\delta _n^{-1} L_{\delta _n}$ converges as $n \rightarrow \infty$. Then the claim holds for $c^+ = \lim _{n \rightarrow \infty } \delta _n^{-1} L_{\delta _n}$. $\quad \square$

##### Proposition 4.4

For any sequence $\delta _n \searrow 0$ as $n \rightarrow \infty$, any sequence $\mathbb {H}_{\delta }^{(z_0, \theta )}$ (where $z_0$, $\theta$ are fixed, for simplicity) and any $r>0$, there exists a subsequence $\delta _{n_k}$ and constants $c \in \mathbb {R}_{>0}$ and $\beta \in \mathbb {R}_{\ge 0}$ such that for any sequence of domains $\Omega _{\delta _n}$ that agrees with $\mathbb {H}_{\delta _n}^{(z_0, \theta )}$ in the *r*-neighborhood of $z_0$ and that converges to a domain $\Omega$ in the Carathéodory sense, the sequence $\delta _{n_k}^{-1} H_{\delta _{n_k}}$ converges as $k \rightarrow \infty$ uniformly on compact sets to a harmonic function *h* that satisfies the following boundary conditions

45 $$\begin{aligned} h={\left\{ \begin{array}{ll} c \beta &{} \text {on } (a\,b) \\ \text {bounded} &{} \text {near } a \text { and } b \\ 0 &{} \text {on } (b\, z_0) \text { and } (z_0\, a) \\ c {{\,\mathrm{Im}\,}}( -\frac{e^{i \theta }}{z-z_0}) + \mathcal {O}(1) &{} \text {as } z \rightarrow z_0 \end{array}\right. } \end{aligned}$$

Furthermore, $\delta _{n_i}^{-1} H^+_{\delta _{n_i}}$ converges uniformly on compact sets to $c^+ {{\,\mathrm{Im}\,}}( -\frac{e^{i \theta }}{z-z_0})$ along the same subsequence and $c=c^+$ and the convergence is uniform over all domains $\Omega$.

##### Proof

Use the same argument as in the proof of Lemma 4.3 (ii) to show that $\delta _{n_i}^{-1} H_{\delta _{n_i}}$ converges uniformly on compact sets to a harmonic function *h* and it satisfies (45). Suppose that $c \ne c_+$. Then, because *f* can be recovered from *h* by the formula $f = \sqrt{2 i \, \partial _z h}$, it is straightforward to show that

46 $$\begin{aligned} \tilde{h} = {{\,\mathrm{Im}\,}}\int \big (f-f^+\big )^2 = \big (\sqrt{c} - \sqrt{c^+}\big )^2 \frac{{{\,\mathrm{Im}\,}}((z-z_0) \, e^{-i\theta })}{|z-z_0|^2} + \mathcal {O}({{\,\mathrm{Im}\,}}((z-z_0) \, e^{-i\theta })) \end{aligned}$$

as $z \rightarrow z_0$. There exists $r>0$ such that $\tilde{h}$ is positive in $\mathbb {H}^{(z_0, \theta )} \cap B(z_0,r)$.

Next we notice that $\tilde{h}$ is not bounded in $\mathbb {H}^{(z_0, \theta )} \cap B(z_0,r)$. On the other hand, if we consider the discrete version $\tilde{h}_\delta$ of $\tilde{h}$, it must remain bounded on $\mathbb {H}_\delta ^{(z_0, \theta )} \cap \partial B(z_0,r)$ uniformly in $\delta$ because of the convergence to $\tilde{h}$. Thus it is bounded inside $\mathbb {H}_\delta ^{(z_0, \theta )} \cap B(z_0,r)$ because of its 0 boundary values on the straight part of the boundary around $z_0$. This leads to a contradiction. $\quad \square$

##### Remark 4.5

If we normalize by the value of $H^+$, the convergence holds for the whole sequence, not just along subsequences.

##### Remark 4.6

This proof can be generalized to any domain with $z_0$ lying on a smooth boundary segment, if the domain is approximated in a nice way around $z_0$. This means that the boundary near $z_0$ lies between two copies of the same smooth curve shifted by a bounded number of lattice steps, for instance. The upper and lower bound for the harmonic function with a pole at a boundary vertex, evaluated at fixed interior point take the form $const. \delta$ since we can bound it using hitting probabilities in regular regions such as squares or discs (or rather their discrete approximations). The constant depends on the chosen interior point as well as the continuum domain, but not on its discrete approximation. Namely, the local geometry of the boundary near the pole doesn’t play a role as we can take minimums and maximums over the finite number of possibilities (when the boundary is between two curves which are closer than a finite multiple of $\delta$). All *H* functions on domains that have a given *r* neighborhood of $z_0$ must have the same singular part. Moreover, the value at a fixed point of any fixed *H* function can be used for normalization for the other functions and they converge to a limit.

The generalization to non-smooth boundary would require a matching pair of upper and lower bounds for $H_{\delta }$, the former for the restriction to $\mathbb {L}^\circ$ and the latter to $\mathbb {L}^\bullet$. This is therefore equivalent of knowing (uniform bounds for) the leading term of the asymptotics of the exit probabilities with different lattice “boundary shapes” as $\delta \rightarrow 0$. The asymptotics is heavily influenced by the local geometry around $z_0$.

In the final result of this subsection, we derive a characterizing property for the constant $\beta$. Hence this constant is uniquely determined by the continuum setting and doesn’t depend on the discrete approximations we are using. In fact, it only depends on the conformal type of the domain $(\Omega ,a,b,z_0)$ with a prescribed length scale (derivative) at $z_0$.

Suppose for simplicity that $\Omega$ is a Jordan domain. Let $h: \Omega \rightarrow \mathbb {R}$ be a harmonic function, for instance, *h* is the scaling limit from Proposition 4.4. Suppose that *z* is a boundary point and $h= \beta$ near *z* on the boundary. We define a weak version of the sign of the normal derivative $\partial _n h(z)$ to the direction of the outer normal at a boundary point *z* of a (possibly non-smooth) domain by

- $$\partial _n h \ge 0$$ at $$w \in ab$$, if
  $$\begin{aligned} h^{-1}( \,(-\infty ,\beta ]\, ) \cap B(w,\varepsilon ) \ne \emptyset \end{aligned}$$
  for all $$\varepsilon >0$$.
- $$\partial _n h \le 0$$ at $$w \in bc \cup ca$$, if
  $$\begin{aligned} h^{-1}( \,[\beta ,\infty )\, ) \cap B(w,\varepsilon ) \ne \emptyset \end{aligned}$$
  for all $$\varepsilon >0$$.

##### Proposition 4.7

The constant $\beta$ in (45) is the unique constant such that *h* has the normal derivatives

47 $$\begin{aligned} {\left\{ \begin{array}{ll} \partial _n h \ge 0 &{} \text {on } ab \\ \partial _n h \le 0 &{} \text {on } bc \text { and } ca \end{array}\right. } \end{aligned}$$

and there exists a point *w* on *ab* such that $h^{-1}( \,(\beta ,\infty )\, ) \cap B(w,\varepsilon ) \ne \emptyset$ for all $\varepsilon >0$.

##### Proof

The normal derivatives (47) follow by the same argument as in [10], Section 6.

Suppose that $\phi :U \rightarrow \mathbb {H}$ is conformal and onto and $\phi (c)=\infty$. Then $h = h^\mathbb {H}\circ \phi$ where

48 $$\begin{aligned} h^\mathbb {H}(z) = {{\,\mathrm{Im}\,}}\int (f^\mathbb {H})^2 \end{aligned}$$

and up to a universal multiplicative constant

49 $$\begin{aligned} f^\mathbb {H}(z)= \sqrt{1 + \beta \left( -\frac{1}{z-u} + \frac{1}{z-v}\right) }, \end{aligned}$$

where $$u=\phi (a)$$ and $$v=\phi (b)$$.

Write

50 $$\begin{aligned} f^\mathbb {H}(z)= \sqrt{\frac{Q(z)}{(z-u)(z-v)}}, \end{aligned}$$

where

51 $$\begin{aligned} Q(z)=z^2 - (u+v) z + (uv + \beta (v-u)) . \end{aligned}$$

Since the coefficients of the quadratic polynomial *Q* are real, it either has two real zeros or a pair of complex conjugate zeros (with non-zero imaginary part). Since (50) holds and $f^\mathbb {H}$ is single-valued in $\mathbb {H}$ (being a scaling limit of a discrete observable which is single-valued), there can’t be any zeros of multiplicity one in the upper half-plane. Thus the zeros of *Q* are real.

Now

52 $$\begin{aligned} \partial _n h^\mathbb {H}(x) = - \partial _y h^\mathbb {H}(x) = -{{\,\mathrm{Re}\,}}[(f^\mathbb {H}(x))^2] = \frac{Q(x)}{(x-u)(v-x)} \end{aligned}$$

on $\mathbb {R}{\setminus } \{u,v\}$. Let the zeros of *Q* be $w_1$ and $w_2$ with $w_1 \le w_2$. Then $w_1 + w_2 = u+v$ by (51). Therefore we have to analyze the following four cases

1. $$u< w_1=w_2 < v$$
2. $$u< w_1< w_2 < v$$
3. $$u=w_1 < w_2=v$$
4. $$w_1< u< v < w_2$$.

We notice using (52) that only the first case is consistent with (47).

Thus we have shown that $w_1=w_2 = (u+v)/2$ and hence $\beta = (v-u)/4$. The normal derivative $\partial _n h^\mathbb {H}(x)$ is positive when $x \in (u,v)$ and negative when $x \in \mathbb {R}{\setminus } [u,v]$. The value of $\beta$ is uniquely determined and it is the only real value such that $\partial _n h$ has the properties claimed. $\quad \square$

### Value of $\beta$ for the 4-point observable

We will determine the value of $\beta$ for the 4-point observable in the same way as in Proposition 4.7. Remember that $0 \le \beta \le 1$.

Consider $f^\mathbb {H}$ for the domain $\mathbb {H}$ with the four marked boundary points $u<v<w$ and $\infty$. This means that *h* can be written as $h = h^\mathbb {H}\circ \phi$ where

53 $$\begin{aligned} h^\mathbb {H}= {{\,\mathrm{Im}\,}}\int (f^\mathbb {H})^2 \end{aligned}$$

and $$\phi (a)=u$$, $$\phi (b)=v$$, $$\phi (c)=w$$ and $$\phi (d)=\infty $$.

For any $$u<v<w$$

54 $$\begin{aligned} h^{\mathbb {H},u,v,w} (z) = \frac{1}{\pi } {{\,\mathrm{Im}\,}}\left( -\log (z-w) + \beta \left( -\log (z-u) + \log (z-v) \right) \right) . \end{aligned}$$

Hence

55 $$\begin{aligned} \sqrt{\pi } \, f^{\mathbb {H},u,v,w} (z)&= \sqrt{ -\frac{1}{z-w} + \beta \left( -\frac{1}{z-u} + \frac{1}{z-v} \right) } \nonumber \\&= \sqrt{ -\frac{ Q(z)}{(z-u)(z-v)(z-w)} } \end{aligned}$$

where *Q*(*z*) is a quadratic polynomial.

Let’s simplify things by setting $u=0$ and $w=1$. Hence for $0<v<1$, *Q*(*z*) can be written as

56 $$\begin{aligned} Q(z)&= z\,(z-v) - \beta \,v\, (z-1) \nonumber \\&= z^2 - (\beta +1)\,v\,z + \beta \,v . \end{aligned}$$

Since the coefficients of *Q* are real, there are two options: *either* we have that ${{\,\mathrm{Im}\,}}w_1 \ne 0$ or ${{\,\mathrm{Im}\,}}w_2 \ne 0$, and then $w_1^* = w_2$, *or* we have that $w_1$ and $w_2$ are real. Since (55) holds and $f^\mathbb {H}$ is single valued in $\mathbb {H}$, there can’t be any zeros in the upper half-plane. Hence the zeros are real.

Let’s write also in this case the normal derivative in the direction of the outer normal

57 $$\begin{aligned} \partial _n h^\mathbb {H}(x) = -\partial _y {{\,\mathrm{Im}\,}}\int (f^\mathbb {H})^2 = {{\,\mathrm{Re}\,}}\frac{ Q(x)}{x(x-v)(x-1)} \end{aligned}$$

for all $x \in \mathbb {R}{\setminus } \{0,v,1\}$. By the same argument as in [10], Section 6, this normal derivative is negative on $(-\infty ,0) \cup (v,1)$ and positive on $(0,v)\cup (1,\infty )$. This is only possible if the two roots of *Q* are equal. Therefore in addition to $0< \beta < 1$, the constant $\beta$ has to satisfy

58 $$\begin{aligned} (\beta +1)^2 v^2 = 4 \beta v \end{aligned}$$

and hence $\beta =\beta _-$ or $\beta =\beta _+$ where

$$\begin{aligned} \beta _\pm = \frac{ -v + 2 \pm 2 \sqrt{1-v}}{v}. \end{aligned}$$

Let’s write $\beta _\pm -1 = \frac{2}{v} \,\sqrt{1-v}\,(\sqrt{1-v} \pm 1)$, which is positive for $\beta _+$ and negative for $\beta _-$ for all $v \in (0,1)$. Thus $\beta = \beta _-$ and we find that

59 $$\begin{aligned} \beta = \frac{ -v + 2 - 2 \sqrt{1-v}}{v} = \left( \frac{1-\sqrt{1-v}}{\sqrt{v}} \right) ^2 = \left( \tan \left( \frac{x}{2} \right) \right) ^2 \end{aligned}$$

where $0<x<\pi /2$ is such that $v=\sin ^2 x$. Notice that the double root of *Q* is $\sqrt{\beta v}$ and it lies in the interval (0, *v*).

To conclude, we state that the value of $\beta$ is characterized uniquely by the formula (54), the fact that $\beta \in (0,1)$ and that the normal derivative of $h^\mathbb {H}$ is negative on $(-\infty ,u) \cup (v,w)$ and positive on $(u,v)\cup (w,\infty )$.

### A remark on crossing probabilities

As a side remark, let’s derive the probability *P* of the internal arc pattern

60 $$\begin{aligned} (0 \frown \infty , 1 \frown v) \end{aligned}$$

under the random cluster measure where there isn’t any exterior connections, that is, the arcs $\gamma _1$ and $\gamma _2$ (as defined in Sect. 4.1) are not counted as loops in the weight of a configuration. Since $\sqrt{\beta }$ and $1-\sqrt{\beta }$ are proportional to *P* and $\sqrt{2}(1-P)$, respectively, *P* satisfies

61 $$\begin{aligned} \frac{P}{P+\sqrt{2}(1-P)} = \sqrt{\beta } \end{aligned}$$

and hence

62 $$\begin{aligned} P = \frac{\sqrt{2} \sqrt{\beta }}{(\sqrt{2}-1) \sqrt{\beta } + 1 } = \frac{ \sin \left( \frac{x}{2} \right) }{\sin \left( \frac{x}{2} \right) + \sin \left( \frac{\pi }{4} - \frac{x}{2} \right) } . \end{aligned}$$

By using the relations

63 $$\begin{aligned} \sin \left( \frac{x}{2} \right) = \frac{1}{\sqrt{2}} \sqrt{1 - \sqrt{1-v}}, \quad \sin \left( \frac{\pi }{4} - \frac{x}{2} \right) = \frac{1}{\sqrt{2}} \sqrt{1 - \sqrt{v}} \end{aligned}$$

we can write this into the form

64 $$\begin{aligned} P = \frac{ \sqrt{1 - \sqrt{1-v}}}{ \sqrt{1 - \sqrt{1-v}} + \sqrt{1 - \sqrt{v}}} . \end{aligned}$$

This is consistent with the result in [10].

## Characterization of the Scaling Limit

### Martingales and uniform convergence with respect to the domain

Consider the scaling limit of a single branch from *a* to $z_0$ in the domain $\Omega$. To simplify the setting, map the discrete random curves to a reference domain $\mathbb {D}$ using conformal maps so that the resulting probability law $\mathbb {P}_\delta$ is the law of a random curve in $\mathbb {D}$ from $-1$ to 1 and $\mathbb {P}^*$ is any subsequent scaling limit of $\mathbb {P}_\delta$. Consider some scheme of parametrizing the curves that works for all $\delta$. We will mostly use the half-plane capacity parametrization. Let *X* be the space of continuous function from the time interval used for parametrization to $\overline{\mathbb {D}}$. We consider *X* as a metric space with the metric defined by the sup norm. Denote by $\mathcal {F}_t$ the filtration generated by the curve up to time *t*.

After we start exploring the branch we will move automatically from the setting of two points to a setting of three points. Hence we will also consider the setups of $(\Omega ,a,b,z_0)$, where $z_0$ is the fused arc (*cd*), and $(\Omega ,a,b,c,d)$.

Remember that the two martingales were

65 $$\begin{aligned} M_t^{(\delta )}&= \pm \sqrt{\frac{1}{\delta } \beta _t^{(\delta )}} = \pm \sqrt{\frac{1}{\delta } \beta ^{(\delta )}(\Omega _t,a_t,b_t,z_0)} \end{aligned}$$

66 $$\begin{aligned} N_t^{(\delta )}&= \frac{1}{\delta } f_t^{(\delta )}(w_0) = \frac{1}{\delta } f_t^{(\delta ),\Omega _t,a_t,b_t,z_0}(w_0). \end{aligned}$$

Notice that we have included the scaling by a power of $\delta$ that makes these quantities converge in the limit $\delta \rightarrow 0$, at least for subsequences.

Consider one of the processes above, for instance, $(M_t^{(\delta )})_{t \ge 0}$ the martingale property could be formulated so that if $0 \le s < t$ and if $\psi : X \rightarrow \mathbb {R}$ is bounded, uniformly continuous and $\mathcal {F}_s$-measurable, then

67 $$\begin{aligned} \mathbb {E}^{(\delta ), \Omega , a,b,z_0} [ \psi \,M_t^{(\delta )} ] = \mathbb {E}^{(\delta ), \Omega , a,b,z_0} [ \psi \,M_s^{(\delta )} ]. \end{aligned}$$

Now due to the uniform convergence of $\beta ^{(\delta )}(\Omega _t,a_t,b_t,z_0)$ over the domains, Proposition 4.4, the expected values on both sides will converge and we get

68 $$\begin{aligned} \mathbb {E}^{*, \Omega , a,b,z_0} [ \psi \,\tilde{M}_t ] = \mathbb {E}^{*, \Omega , a,b,z_0} [ \psi \,\tilde{M}_s ] \end{aligned}$$

where $\tilde{M}_t = \lim _{n \rightarrow \infty } M_t^{(\delta _n)} = \pm \sqrt{\beta (\Omega _t,a_t,b_t,z_0)}$. Thus $(\tilde{M}_t)_{t \ge 0}$ is a martingale.

By a similar argument, $\tilde{N}_t = \lim _{n \rightarrow \infty } N_t^{(\delta _n)}= f_t^{\Omega _t,a_t,b_t,z_0}(w_0)$ defines a martingale.

### Simple martingales and a martingale problem

We wrote *f* in the upper half-plane already in (49). Let us now analyze what happens for a growing curve which we interpret as a random Loewner chain. For that we use Theorem 3.12. Next we notice that for all domains (and their approximating sequences) that agree near $z_0$, we had a singularity in *H* with the same constant in front, see Proposition 4.4. Fix some domain $(\Omega ,a,b,z_0)$ and map it to the upper half-plane conformally. Suppose that *w* is the image of $z_0$. Then the singularity is of the form $c {{\,\mathrm{Im}\,}}( -1/(z-w) )$. If we have a slit domain $(\Omega {\setminus } \gamma [0,t],\gamma (t),b,z_0)$ and we apply further the Loewner map $g_t$ in the upper half-plane, then the singularity has to be $c {{\,\mathrm{Im}\,}}( -g_t^{\prime }(w)/(g_t(z)-g_t(w)) ) = c {{\,\mathrm{Im}\,}}( -1/((z-w) ) + o(1)$, as $z \rightarrow w$. This shows that the functions *H* transform as

69 $$\begin{aligned} H^{\mathbb {H}{\setminus } K_t ,U_t,V_t,w} (z) = g_t^{\prime }(w) H^{\mathbb {H},g_t(U_t),g_t(V_t),g_t(w)} (g_t(z)) . \end{aligned}$$

Since $f= \sqrt{ 2i \Phi ^{\prime }}$ where $\Phi$ is holomorphic and ${{\,\mathrm{Im}\,}}\Phi = H$,

70 $$\begin{aligned} f^{\mathbb {H}{\setminus } K_t ,U_t,V_t,w} (z) = \sqrt{g_t^{\prime }(w) \, g_t^{\prime }(z)} f^{\mathbb {H},g_t(U_t),g_t(V_t),g_t(w)} (g_t(z)) . \end{aligned}$$

Now if we choose to send *w* to $\infty$, then the observable is of the form (49). For Loewner chains $g_t^{\prime }(\infty )=1$ when appropriately interpreted, and hence

71 $$\begin{aligned} f^{\mathbb {H}{\setminus } K_t ,U_t,V_t,\infty } (z) = \sqrt{g_t^{\prime }(z)} \, \sqrt{ 1 + \beta _t \left( -\frac{1}{g_t(z)-U_t} + \frac{1}{g_t(z)-V_t} \right) }. \end{aligned}$$

As we saw in the proof of Proposition 4.7, the value of $\beta _t$ is

$$\begin{aligned} \beta _t = \frac{1}{4} (V_t - U_t) . \end{aligned}$$

We define

72 $$\begin{aligned} M_t&= \pm \sqrt{4\beta _t} = \pm \sqrt{V_t - U_t} \end{aligned}$$

73 $$\begin{aligned} N_t&= 4\big (\beta _t (V_t-U_t) - 2t\big ) = M_t^4 - 8t . \end{aligned}$$

The former quantity is proportional to $\tilde{M}_t$ and the latter one to the first non-trivial coefficient in the expansion of (71) around $z=\infty$. Here ± signs are constant on each excursion of $V_t - U_t$ and distributed as independent fair (symmetric) coin flips for each excursion. Here we interpret that an excursion starts at 0, ends at 0 and is positive in between.

By the martingale properties in Sect. 4.1 and the convergence results of the observables we have the following result.

#### Proposition 5.1

Let $\mathbb {P}^*$ be a subsequent limit of the sequence of laws of FK Ising branch in discrete approximations of $(\Omega ,a,b,z_0)$. Let $\phi : \Omega \rightarrow \mathbb {H}$ be a conformal, onto map such that $\phi (z_0)=\infty$. Let $\gamma$ be the random curve distributed according to $\mathbb {P}^*$ in the capacity parametrization, $U_t = \phi (\gamma (t))$ and $V_t$ is the “right-most point” in the hull of $\phi (\gamma (t))$. Let the signs in (72) be i.i.d. fair coin flips independent of $\gamma$. Then processes $(M_t)_{t \ge 0}$ and $(N_t)_{t \ge 0}$ are martingales.

In the rest of this section we consider the following *martingale problem*:

Let $(U_t,V_t)_{t \ge 0}$, $(M_t)_{t \ge 0}$ and $(N_t)_{t \ge 0}$ as above, that is, satisfying that $(M_t)_{t \ge 0}$ and $(N_t)_{t \ge 0}$ are martingales and satisfy relation (73). What is their law given that $(M_t)_{t \ge 0}$ and $(N_t)_{t \ge 0}$ are martingales?

We claim that the required properties (with the above functional dependency of the processes) uniquely determine the joint law of the processes. We call the verification of this claim and the explicit formulation of the law as the *solution of the martingale problem*.

The solution is divided into two part. In Sect. 5.3 we will show that $(|M_t|^{\frac{1}{\alpha }})_{t \ge 0}$ for some $\alpha >0$ is a Bessel process. In Sect. 5.4, we will show that $(V_t)_{t \ge 0}$ follows an evolution such that $V_t$ is a sum of a term from Loewner equation and a term whose value is changing only in the random Cantor set $\{ t \,:\, U_t=V_t\}$, and then we show that the latter “singular” term is in fact identically 0.

### Characterization of $V_t - U_t$

In this section, we show how the “martingale problem” characterizes the law of $(V_t- U_t)$.

More concretely, we work towards the following theorem. Its proof is given in Sect. 5.3.3.

#### Theorem 5.2

Let $X_t = V_t - U_t$ where $U_t$ and $V_t$ are the processes followed by the marked points for the subsequent scaling limit of the FK Ising exploration process. Then $(X_t)_{t \ge 0}$ is a Bessel process of dimension $\delta = 3/2$ scaled by a constant $\sqrt{16/3}$.

#### Remark 5.3

In other words, $(X_t)_{t \ge 0}$ satisfies

74 $$\begin{aligned} \mathrm {d}X_t = \frac{\kappa (\delta -1)}{2 X_t } \mathrm {d}t + \sqrt{\kappa } \mathrm {d}B_t \end{aligned}$$

where $$\kappa = \sqrt{16/3}$$ and $$\delta = 3/2$$.

#### Relation to Lévy’s and Stroock–Varadhan martingale characterizations.

The argument which we will present can be compared to Paul Lévy’s characterization of Brownian motion. The law of Brownian motion $(B_t)_{t \ge 0}$ is characterized by the fact that $B_t$ and $B_t^2 -t$ are martingales. In the setting of general diffusions, the classical Stroock-Varadhan martingale problem approach describes weak solutions $X_t$ to stochastic differential equations of the form $\mathrm {d}X_t = \sqrt{a} \,\mathrm {d}B_t + b \,\mathrm {d}t$ (with coefficients *a* and *b* satisfying suitable measurability conditions) as exactly those that all the quantities of the form $f(X_t) - \int _0^t ( \frac{1}{2} a_s (X) f^{\prime \prime }(X_s) + b_s (X) f^{\prime }(X_s) ) \mathrm {d}s$ are martingales for a class of test functions *f*, see Sections V.19 and V.20 in [19]. However, similarly to Lévy’s theorem, there are stronger results, stating that two (well-chosen) martingales are enough to characterize the law of a diffusion, see [2, 27]. We take this path, using two martingales to show that the diffusion in question is the Bessel process.

#### Lemmas.

We need the next two lemmas, which we write in greater generality suitable for the 4-point case.

##### Lemma 5.4

Suppose that $T>0$ is a stopping time and suppose that $\psi \in C^2$ satisfies $\psi (0)=0$ and $\psi ^{\prime \prime }(0)=0$. If $A_t$ and $C_t$ are continuous, predictable processes which satisfy

1. $A_t$ is of bounded total variation
2. $C_t$ is non-decreasing, differentiable and satisfies $\dot{C}_t>0$ almost surely on [0, *T*).

then any continuous martingale $(M_t)_{t \in [0,T]}$ with the property that the process $(N_t)_{t \in [0,T]}$ defined by

75 $$\begin{aligned} N_t = A_t \psi (M_t) - C_t \end{aligned}$$

is a martingale, satisfies

76 $$\begin{aligned} \mathbb {P}\left[ \int _0^T \mathbbm {1}_{M_t=0} \, \mathrm {d}t =0 \right] = 1. \end{aligned}$$

##### Proof

Since $(M_t)_{t \in [0,T]}$ is a continuous martingale, it has quadratic variation process $\langle M \rangle _t$. By Itô’s formula

77 $$\begin{aligned} \mathrm {d}N_t = \psi (M_t) \,\mathrm {d}A_t - \dot{C}_t \,\mathrm {d}t + \frac{1}{2} A_t \psi ^{\prime \prime }(M_t) \,\mathrm {d}\langle M \rangle _t + A_t \psi ^{\prime }(M_t) \,\mathrm {d}M_t . \end{aligned}$$

Since $\psi (M_t)$ and $\psi ^{\prime \prime }(M_t)$ vanish when $M_t=0$, it follows that

78 $$\begin{aligned} \int _0^t \mathbbm {1}_{M_s=0} \,\mathrm {d}N_s - \int _0^t \mathbbm {1}_{M_s=0} A_s \psi ^{\prime }(M_s) \,\mathrm {d}M_s = - \int _0^t \mathbbm {1}_{M_s=0} \dot{C}_s \,\mathrm {d}s . \end{aligned}$$

The process $\mathbbm {1}_{M_t=0}$ is predictable, because it is a pointwise limit of adapted continuous processes (for instance, $\mathbbm {1}_{M_t=0}=\lim _{n \rightarrow \infty } \max \{0, 1 - n |M_t|\}$), and hence the left-hand side of (78) is a local martingale. On the other hand the right-hand side of (78) is bounded variation process. Hence $\int _0^t \mathbbm {1}_{M_s=0} \dot{C}_s \,\mathrm {d}s =0$ almost surely. Since $\dot{C}_s>0$, the claim follows. $\quad \square$

##### Lemma 5.5

If $(M_t)_{t \in [0,T]}$ is a continuous martingale with respect to $(\mathcal {F}_t)_{t \in [0,T]}$ such that

1. almost surely $$\int _0^T \mathbbm {1}_{M_t=0} \mathrm {d}t = 0$$
2. $\mathrm {d}\langle M \rangle _t = \sigma _t^2 \mathrm {d}t$ and $\sigma _t >0$ for any *t* such that $M_t \ne 0$,

then $(B_t)_{t \in [0,T]}$ defined as

79 $$\begin{aligned} B_t = \int _0^t \mathbbm {1}_{ M_s \ne 0 } \, \sigma _s^{-1} \, \mathrm {d}M_s \end{aligned}$$

is well-defined and continuous and it is a one-dimensional standard $(\mathcal {F}_t)$-Brownian motion.

##### Proof

Let $F= [0,T] {\setminus } \{ t \,:\, M_t=0\}$ which is relatively open in [0, *T*]. For any $(t_{k-1},t_k] \subset F$, it holds that $\int \mathbbm {1}_{(t_{k-1},t_k]} \,\sigma _s^{-2} \,\mathrm {d}\langle M \rangle _s = t_k-t_{k-1}$ by the assumptions. We can approximate the set *F* from below by finite unions of this type of intervals and use monotone convergence theorem to show that

80 $$\begin{aligned} \int _0^t \mathbbm {1}_F \,\sigma _s^{-2} \,\mathrm {d}\langle M \rangle _s = \int _0^t \mathbbm {1}_F \,\mathrm {d}s \end{aligned}$$

where the left-hand side is a Lebesgue–Stieltjes integral and the right-hand side is a Lebesgue integral defined pointwise in the randomness almost surely.

This shows that $\mathbbm {1}_F \sigma _s^{-1}$ is $\mathrm {d}M_s$ integrable (belongs to the square integrable processes with respect to the variation process of *M*) and $(B_t)$ is well-defined and continuous in *t*. Therefore clearly, $(B_t)$ is a local martingale and satisfies $\langle B \rangle _t = t$. Hence $(B_t)$ is a standard Brownian motion by Lévy’s characterization theorem. $\quad \square$

#### $X_t=V_t - U_t$ is a Bessel process.

##### Proof of Theorem 5.2

The claim follows from the next theorem with $M_t = \frac{1}{2} \sqrt{X_t}$, or written in the other way $X_t = 4 M_t^2$, and $\psi (x)= x^4$. Notice that if $C M_t^4$, where $C>0$ is a constant, is a squared Bessel process of dimension $\delta$, then $(\sqrt{C}/4) X_t$ is a Bessel process of dimension $\delta$. Here $\delta = 3/2$ and $C=2 \delta = 3$, which implies that $X_t$ is a Bessel process scaled by the constant $4/\sqrt{C} = \sqrt{16/3}$. $\quad \square$

##### Theorem 5.6

Let $(a,b) \subset \mathbb {R}$. Suppose that $\psi : (a,b) \rightarrow \mathbb {R}$ is twice continuously differentiable function which is convex, i.e. $\psi ^{\prime \prime } \ge 0$, and such that $\psi ^{\prime }$ is strictly increasing. Let $M=(M_t)_{t \in \mathbb {R}_+}$ be a continuous stochastic process adapted to a filtration $(\mathcal {F}_t)_{t \in \mathbb {R}_+}$ and $N=(N_t)_{t \in \mathbb {R}_+}$ a process defined by $N_t = 2 \psi (M_t) - t$. Suppose that *M* and *N* are martingales. Then the following claims hold.

1. If the process $W=(W_t)_{t \in \mathbb {R}_+}$ is defined as
  $$\begin{aligned} W_t = \int _0^t \sqrt{\psi ^{\prime \prime }(M_s)} \mathrm {d}M_s , \end{aligned}$$
  then it is a standard Brownian motion.
2. If for some $\varepsilon >0$, $\psi ( x ) = |x|^{2 + \varepsilon }$, then there exists constants $C>0$ and $1<\delta <2$ such that $Z_t = C \, \psi (M_t)$ is a squared Bessel process of dimension $\delta$. More specifically, the constants are $\delta = 2 \frac{1+\varepsilon }{2+\varepsilon }$ and $C=2 \delta$.
3. Suppose there exists continuous functions $F,\tilde{F}$ such that $2\tilde{F}(x)=F(2 \psi (x))$ and $\psi ^{\prime }(x) = \,\mathrm {sgn}(\psi ^{\prime }(x)) \tilde{F}(x) \sqrt{\psi ^{\prime \prime }(x)}$ for all *x* — in particular $\psi ^{\prime \prime }$ is positive except possibly at the point (there exists at most one such point) where $\psi ^{\prime }$ is zero. Then $Z_t = 2\psi (M_t)$ is a solution to the stochastic differential equation
  81 $$\begin{aligned} \mathrm {d}Z_t = F(Z_t) \mathrm {d}\tilde{W}_t + \mathrm {d}t \end{aligned}$$
  for some standard Brownian motion $(\tilde{W}_t)_{t \in \mathbb {R}_+}$.

##### Proof

1. Similarly as in the previous section, we notice that we can do stochastic analysis with *M*, because *M* is a continuous martingale, see Chapter 2 of [12]. The same argument, using that $(M_t)_{t \in \mathbb {R}_+}$ and $(N_t)_{t \in \mathbb {R}_+}$ are martingales and that $N_t$ is given in terms of $M_t$ as $N_t = 2 \psi (M_t) - t$, as above tells us the process defined as
  $$\begin{aligned} W_t = \int _0^t \sqrt{\psi ^{\prime \prime }(M_s)} \,\mathrm {d}M_s , \end{aligned}$$
  is a continuous martingale with a variation process $\langle W \rangle _t = t$. Namely, by Itô’s lemma we have $\mathrm {d}N_t = (\psi ^{\prime \prime }(M_t)\mathrm {d}\langle M \rangle _t - \mathrm {d}t) + 2\psi ^{\prime }(M_t) \mathrm {d}M_t$ and thus by martingale property of $N_t$ the quantity inside the first brackets has to vanish identically, and we can apply the previous lemmas for the claim. Hence by Lévy’s characterization theorem, it is a standard Brownian motion.
2. When $\psi ( x ) = |x|^{2 + \varepsilon }$, there is a constant $D>0$ such that
  $$\begin{aligned} \psi ^{\prime }(x) = D \,\mathrm {sgn}(x) \sqrt{ \psi ^{\prime \prime }(x) \psi (x) }. \end{aligned}$$
  Therefore $$Z_t = 2 \tilde{C} \psi (M_t)$$ satisfies
  $$\begin{aligned} \mathrm {d}Z_t&= 2 C \psi ^{\prime }(M_t) \,\mathrm {d}M_t + \tilde{C} \psi ^{\prime \prime }(M_t) \mathrm {d}\langle M \rangle _t \\&= 2 \tilde{C}\, D \,\mathrm {sgn}(M_t) \sqrt{ \psi (M_t) } \,\mathrm {d}W_t + \tilde{C} \mathrm {d}t \\&= 2\, \sqrt{\tilde{C}/2}\, D \,\mathrm {sgn}(M_t) \sqrt{ Z_t } \,\mathrm {d}W_t + \tilde{C} \mathrm {d}t . \end{aligned}$$
  Hence if we choose $\tilde{C}= 2 \, D^{-2}$, $Z_t$ is squared Bessel process with the parameter $\delta = 2\, D^{-2}$. Here we used the fact that $\int _0^t \,\mathrm {sgn}(M_s) \,\mathrm {d}W_s$ is a standard Brownian motion by Lévy’s characterization theorem. The claim follows for $C=2 \tilde{C}$. A direct calculation shows that $D= \sqrt{(2+\varepsilon )/(1+\varepsilon )} \in (1,\sqrt{2})$.
3. Similarly as above we can write
  $$\begin{aligned} \mathrm {d}Z_t&= 2 \psi ^{\prime }(M_t)\,\mathrm {d}M_t + \psi ^{\prime \prime }(M_t) \,\mathrm {d}\langle M \rangle _t \\&= 2 \,\mathrm {sgn}(\psi ^{\prime }(M_t)) \tilde{F}(M_t) \, \mathrm {d}W_t + \mathrm {d}t \\&= \,\mathrm {sgn}(\psi ^{\prime }(M_t)) F(Z_t) \, \mathrm {d}W_t + \mathrm {d}t \end{aligned}$$
  from which the claim follows.$\quad \square$

##### Remark 5.7

Note that when $1/\alpha =2+\varepsilon$ and $\alpha = 1 - \delta /2$ and $D= \sqrt{(2+\varepsilon )/(1+\varepsilon )}$, then $2\, D^{-2} = \delta$.

### Characterization of $(U_t,V_t)$

Next result together with Theorem 5.2 gives the distribution of the pair of processes $(U_t,V_t)$.

#### Theorem 5.8

Let $X_t,V_t,U_t$ be as in Theorem 5.2. Then $U_t$ and $V_t$ satisfy

82 $$\begin{aligned} V_t&= V_0 +2 \int _0^t \frac{\mathrm {d}s}{X_s} \end{aligned}$$

83 $$\begin{aligned} U_t&= U_0 +2 \int _0^t \frac{\mathrm {d}s}{X_s} - X_t + X_0. \end{aligned}$$

#### Remark 5.9

This equation for $V_t$ is the Loewner equation. Notice that $\int _0^t \frac{\mathrm {d}s}{X_s}$ is finite since $(X_t)$ is Bessel process of dimension $\delta = 3/2$. The equation for $U_t$ is obtained from the one of $V_t$ and the definition of $X_t$.

#### Proof of Theorem 5.8

Since $\int _0^t X_s^{-1}\mathrm {d}s$ is finite, we have shown so far that

84 $$\begin{aligned} V_t&= V_0 +2 \int _0^t \frac{\mathrm {d}s}{X_s} + \Lambda _t \end{aligned}$$

85 $$\begin{aligned} U_t&= U_0 +2 \int _0^t \frac{\mathrm {d}s}{X_s} - X_t +X_0 + \Lambda _t \end{aligned}$$

where $\Lambda _t$ is some non-decreasing process which is constant on any subinterval of $\{t \,:\, X_t>0\}$. See Proposition C.2 in Appendix C for the generalized Loewner equation. The claim follows when we show that $\Lambda _t\equiv 0$. This is shown in the next proposition. $\quad \square$

#### Proposition 5.10

$$\Lambda _t \equiv 0$$.

#### Proof

Let $\Sigma = \{t \,:\, X_t=0\}$. The *index* of a Bessel process is defined as

86 $$\begin{aligned} \nu = \frac{\delta }{2}-1 . \end{aligned}$$

Notice that $\nu =-1/4$ in our case when the Bessel process has dimension 3 / 2. By [18] Exercise XI.1.25 and [5] Theorem III.15, the Hausdorff dimension of the support of the local time of a Bessel process with index $\nu \in [-1,0]$ is $-\nu$. Hence the Hausdorff dimension $\dim _\mathcal {H}( \Sigma ) = 1/4$.

Notice next that $(X_t)_{t \in [0,T]}$ is $1/2 - \varepsilon$ Hölder continuous for any $\varepsilon >0$, since it is a Bessel process scaled by constant (this can be derived in several ways; for instance starting from the density of the transition semigroup [18, p. 446] and checking the asumptions of the Kolmogorov continuity theorem) and $(\int _0^t X_s^{-1} \mathrm {d}s)_{t \in [0,T]}$ is $1/2 - \varepsilon$ Hölder continuous for any $\varepsilon >0$, since it can be written as

$$\begin{aligned} \int _0^t X_s^{-1} \mathrm {d}s = \frac{3}{4} \left( X_t - X_0 - \frac{4}{\sqrt{3}} B_t \right) \end{aligned}$$

from (74).

The claim follows from Lemma 5.11 and from the fact that $(U_t)_{t \in [0,T]}$ is $1/2 - \varepsilon$ Hölder continuous for any $\varepsilon >0$ by Theorem 3.12. $\quad \square$

#### Lemma 5.11

Let $I \subset [0,S]$ be a closed set with Hausdorff dimension $\alpha _0 \in [0,1]$. Suppose that $f: [0,S] \rightarrow \mathbb {R}$ is continuous and constant on any subinterval of $[0,S] {\setminus } I$. If *f* is not a constant function and if *f* is Hölder continuous with exponent $\alpha$, then $\alpha \le \alpha _0$.

The proof is standard using the definition of Hausdorff measure and

$$\begin{aligned} |f(x) -f(y)| \le C |x-y|^\alpha , \end{aligned}$$

and we leave it to the industrious reader.

### The martingale characterization in the 4-point case

#### The $\hbox {SLE}[\kappa ,Z]$ process.

The drift of a SLE process can be given in general in terms of a partition function $Z(u,v,\ldots )$ where $u,v,\ldots$ are the marked points of the process (for instance, the chordal SLE has marked points $u, \infty$ and $\infty$ doesn’t appear explicitly in *Z*, and the $\hbox {SLE}({\kappa ,\rho })$ has marked points $u,v,\infty$). We call such process $\hbox {SLE}[\kappa ,Z]$ and the driving process $U_t$ is given by

87 $$\begin{aligned} \mathrm {d}U_t = \sqrt{\kappa }\mathrm {d}B_t + \kappa \partial _u \log Z(U_t,V_t,\ldots ) \mathrm {d}t . \end{aligned}$$

The other points follow the Loewner equation.

The partition function is not unique, but if we require Möbius covariance and finite limit as $w \rightarrow \infty$, then $\hbox {SLE}[\kappa ,Z]$ with $\kappa = 16/3$

88 $$\begin{aligned} Z(u,v,w)= \frac{1}{y^{1/8}\,(m^2+1)^{1/4}\,m^{1/4}} \end{aligned}$$

where $y=w-v$, $m=\sqrt{1+y/x} - \sqrt{y/x}$ and $x=v-u$, describes the scaling limit of FK Ising exploration in $(\Omega ,a,b,c,d)$ from *a* to *d* which is reflected on *bc* towards *d*. Notice that the process with the partition function (88) and $\kappa = 16/3$ isn’t a $\hbox {SLE}({\kappa ,\rho })$ process.

##### Theorem 5.12

In the 4-point setting, the scaling limit of FK Ising exploration in $(\Omega ,a,b,c,d)$ from *a* to *d* which is reflected on *bc* towards *d* is $\hbox {SLE}[\kappa ,Z]$ with $\kappa = 16/3$ and the partition function given by (88).

This result follows from the estimates on Hölder regularity of the random curves and the characterization result Theorem 5.13 below. It is possible to use this result to show that the interface when conditioned on an internal arc pattern $(a \frown d, b \frown c)$ converges towards so called hypergeometric SLE [16].

##### Theorem 5.13

Let $X_t = V_t - U_t$, $Y_t = W_t - V_t$ where $U_t$, $V_t$ and $W_t$ are the processes followed by the marked points for the subsequent scaling limit of the FK Ising exploration process in the 4-point setting. Then for some Brownian motion $(B_t)_{t \ge 0}$ the pair of processes $(X_t, Y_t)_{t \ge 0}$ satisfies

89 $$\begin{aligned} X_t&= X_0 + \frac{4}{\sqrt{3}} \, B_t + \frac{1}{3} \, \int _0^t \frac{(3M_s^4+2M_s^2+1)(1-M_s^2)^2}{Y_s M_s^2 (M_s^2+1)^2} \mathrm {d}s \end{aligned}$$

90 $$\begin{aligned} Y_t&= Y_0 -\int _0^t \frac{1}{2 Y_s} \frac{\left( 1-M_s^2\right) ^4 }{ M_s^2 (1+M_s^2)^2 } \mathrm {d}s \end{aligned}$$

where

91 $$\begin{aligned} M_t^2 = \left( \sqrt{1 + \frac{Y_t}{X_t}} - \sqrt{\frac{Y_t}{X_t}} \right) ^2 . \end{aligned}$$

Furthermore, the driving process $(U_t)_{t \ge 0}$ is recovered from $(Y_t)_{t \ge 0}$ by

92 $$\begin{aligned} U_t = U_0 +2 \int _0^t \frac{\mathrm {d}s}{X_s} - X_t + X_0. \end{aligned}$$

##### Remark 5.14

Notice that (89) is a combination of Loewner equations for $V_t$ and $W_t$ and (92) follows from definition of $X_t$ and the Loewner equation for $V_t$. Notice also that the given form of *Z* in $\hbox {SLE}[\kappa ,Z]$ follows from a direct calculation using the Eq. (87).

We work towards the proof of this result in next subsections.

#### Simple martingales from the observable.

Let $U_t$ be the driving process, $V_t$ the point corresponding to $b_t$, and $W_t$ the point corresponding to *c* then set

93 $$\begin{aligned} X_t&= V_t - U_t \end{aligned}$$

94 $$\begin{aligned} Y_t&= W_t - V_t . \end{aligned}$$

Then given $\gamma [0,t]$ in $\mathbb {H}$, the map

95 $$\begin{aligned} z \mapsto \frac{g_t(z) - U_t}{W_t - U_t} \end{aligned}$$

maps $H_t = \mathbb {H}{\setminus } \gamma [0,t]$ onto $\mathbb {H}$ and the marked points to 0, $X_t/(X_t + Y_t)$ and 1. Therefore

96 $$\begin{aligned} \sqrt{-\pi } \, f^{\mathbb {H},U_t,V_t,W_t} (z) = \sqrt{g_t^{\prime }(z)} \, \sqrt{ \frac{1}{g_t(z)-W_t} + \beta _t \left( \frac{1}{g_t(z)-U_t} - \frac{1}{g_t(z)-V_t} \right) } \end{aligned}$$

where

97 $$\begin{aligned} \beta _t = \beta \left( \frac{X_t}{X_t + Y_t}\right) = \left( \sqrt{1 + \frac{Y_t}{X_t}} - \sqrt{\frac{Y_t}{X_t}} \right) ^2. \end{aligned}$$

The quantity

98 $$\begin{aligned} M_t = \pm \sqrt{ \beta _t} \end{aligned}$$

is a conditional probability on $\mathcal {F}_t$ of the event $\gamma _1 \subset \hat{\gamma }$ hence $(M_t)_{t \ge 0}$ is a martingale. Here ± sign is needed to extend the martingale property beyond the hitting of 0 by $M_t$. We can solve $X_t$ in terms of $Y_t$ and $M_t$ as

99 $$\begin{aligned} X_t = Y_t \, \frac{4 M_t^2}{(1-M_t^2)^2}. \end{aligned}$$

From the expansion of *f* as $z \rightarrow \infty$, we get that

100 $$\begin{aligned} N_t = X_t \, M_t^2 - W_t \end{aligned}$$

is a martingale.

Write (100) as

101 $$\begin{aligned} N_t = Y_t \psi (M_t) - W_t \end{aligned}$$

where $\psi (m) = 4 [ m^2/(1-m^2 ) ]^2$. Note that $W_t$ is always differentiable and $Y_t$ is differentiable outside the set of times $\Sigma \mathrel {\mathop :}= \{ t \,:\, X_t=0\} = \{ t \,:\, M_t=0\}$. Now we have to solve the following *martingale problem*:

for given filtered probability space $(\mathbb {P}^*,(\mathcal {F}_t))$, determine the law of $(M_t,Y_t,W_t)_{t \in [0,T]}$ which satisfies

1. $(W_t)$ is strictly increasing and $C^1$ for all $t \in [0,T]$
2. $(Y_t)$ is strictly decreasing and $C^1$ for all $t \in [0,T] {\setminus } \Sigma$
3. $\dot{W}_t$ is given by
  102 $$\begin{aligned} \dot{W}_t = \frac{2}{Y_t} \left( \frac{1-M_t^2 }{1+M_t^2} \right) ^2 \end{aligned}$$
  and for a non-decreasing process $\Lambda _t$ given by Proposition C.2 in Appendix C, it holds that
  103 $$\begin{aligned} Y_t = Y_0 -\int _0^t \frac{1}{2 Y_s} \frac{\left( 1-M_s^2\right) ^4 }{ M_s^2 (1+M_s^2)^2 } \mathrm {d}s - \Lambda _t . \end{aligned}$$
4. $(M_t)$ and $(N_t)$ [defined by (101)] are martingales.

It is understood that solving the martingale problem means, as before, that we claim that these properties uniquely characterize the law of $(M_t)$ and hence also the law of $(X_t)$ and also that the law can be explicitly descibred.

#### Solving the martingale problem.

By the lemmas of Sect. 5.3.2, we can construct out of $(M_t)$, which is a continuous martingale, a Brownian motion $(B_t)$ so that there exists a process $(\sigma _t)$, $\sigma _t \ge 0$, both adapted to $(\mathcal {F}_t)$, such that

104 $$\begin{aligned} \langle M \rangle _t = \int _0^t \sigma _s^2 \,\mathrm {d}s, \quad M_t = M_0 + \int _0^t \sigma _s \,\mathrm {d}B_s . \end{aligned}$$

Hence we know that there exists a $(\mathbb {P}^*,(\mathcal {F}_t))$ Brownian motion $(B_t)$ and when $M_t \ne 0$, $\mathrm {d}M_t = \sigma _t \mathrm {d}B_t$. Write the drift of the process $(N_t)$ using Itô’s lemma as

105 $$\begin{aligned} \sigma _t^2 \cdot \frac{24 \, Y_t \, M_t^2 (M_t^2+1)}{(M_t^2-1)^4} - \frac{2(M_t^2-1)^2}{Y_t(M_t^2+1)} . \end{aligned}$$

Therefore we have to have

106 $$\begin{aligned} \sigma _t = \frac{1}{2 \sqrt{3}} \frac{(1-M_t^2)^3}{Y_t\,|M_t| (M_t^2+1)} . \end{aligned}$$

Hence the solution of the martingale problem is that

107 $$\begin{aligned} M_t = M_0 + \int _0^t \frac{1}{2 \sqrt{3}} \frac{(1-M_s^2)^3}{Y_s\,|M_s| (M_s^2+1)} \mathrm {d}B_s. \end{aligned}$$

If we plug this into the expression of $X_t = (4 M_t^2)/(Y_t (M_t^2-1)^2)$ which can be solved from (97) and (98), we get

108 $$\begin{aligned} \mathrm {d}X_t = \frac{4}{\sqrt{3}} \mathrm {d}B_t + \frac{1}{3} \frac{(3M_t^4+2M_t^2+1)(M_t^2-1)^2}{Y_t M_t^2 (M_t^2+1)^2} \mathrm {d}t \end{aligned}$$

which is equivalent to (89).

Notice that when $X_t/Y_t \rightarrow 0$ then

109 $$\begin{aligned} M_t \approx \pm \frac{1}{2} \sqrt{ \frac{X_t}{Y_t} } \end{aligned}$$

and (108) becomes

110 $$\begin{aligned} \mathrm {d}X_t \approx \frac{4}{\sqrt{3}} \mathrm {d}B_t + \frac{4}{3} \frac{\mathrm {d}t}{X_t} \end{aligned}$$

which corresponds to the Bessel process with dimension $\delta =\frac{3}{2}$, as it should.

In the same way, when $X_t/Y_t \rightarrow 0$

111 $$\begin{aligned} \sigma _t \approx \frac{1}{\sqrt{3}} \frac{1}{\sqrt{X_t Y_t}} \end{aligned}$$

Hence $\sigma _t^2$ is integrable with respect to $\mathrm {d}t$ in the same sense as $X_t^{-1}$.

##### Lemma 5.15

Processes $(X_t)_{t \in [0,\infty )}$ and $(\int _0^t X_s^{-1} \mathrm {d}s)_{t \in [0,\infty )}$ are Hölder continuous with any exponent less than $\frac{1}{2}$.

##### Proof

This claim follows from comparison to Bessel process (109) and (110) and a similar argument as we used for a similar claim in the proof of Proposition 5.10. $\quad \square$

#### Characterization of $(U_t,V_t,W_t)_{t \in [0,T]}$.

##### Proof of Theorem 5.13

By comparing to a Bessel process as we did in Sect. 5.5.3, we see that $\int _0^t X_s^{-1}\mathrm {d}s$ is finite. Therefore we have shown so far that

112 $$\begin{aligned} V_t&= V_0 +2 \int _0^t \frac{\mathrm {d}s}{X_s} + \Lambda _t \end{aligned}$$

113 $$\begin{aligned} U_t&= V_0 +2 \int _0^t \frac{\mathrm {d}s}{X_s} - X_t + \Lambda _t \end{aligned}$$

where $\Lambda _t$ is some non-decreasing process which is constant on any subinterval of $\{t \,:\, X_t>0\}$. It remains to show that $\Lambda _t \equiv 0$. This implies then that (90) and (92) hold and finalizes the proof of Theorem 5.13.

Recall from the proof of Proposition 5.10 that $\Sigma = \{t \,:\, X_t=0\}$ and that the index of a Bessel process is defined as

114 $$\begin{aligned} \nu = \frac{\delta }{2}-1 . \end{aligned}$$

Notice that $\nu =-1/4$ in our case.

Now $\Lambda _t \equiv 0$ follows from Lemmas 5.11, 5.16 below, which shows that the Hausdorff dimension of $\Sigma$ is 1 / 4, and from the fact that $(U_t)_{t \in [0,T]}$ is $1/2 - \varepsilon$ Hölder continuous for any $\varepsilon >0$ by Lemma 5.15. $\quad \square$

##### Lemma 5.16

$$\dim _\mathcal {H}(\Sigma ) = 1/4$$

##### Proof

For fixed $\varepsilon >0$ choose $\varepsilon _1>0$ such that

115 $$\begin{aligned} \frac{1-\varepsilon }{3} \frac{1}{m^2}\le & {} \frac{1}{3} \frac{(3 m^4+2 m^2+1)(m^2-1)^2}{m^2 (m^2+1)^2} \le \frac{1}{3} \frac{1}{m^2} \end{aligned}$$

116 $$\begin{aligned} \frac{1}{2 (1+\varepsilon )^{1/2} } \sqrt{x}\le & {} \sqrt{1 + \frac{1}{x}} - \sqrt{\frac{1}{x}} \le \frac{1}{2} \sqrt{x} \end{aligned}$$

for all $0< m \le \sqrt{\varepsilon _1}/2$ and $0< x \le \varepsilon _1$. Then whenever *t* is such that $X_t/Y_t \le \varepsilon _1$, it is possible to estimate

117 $$\begin{aligned} \frac{4(1-\varepsilon )}{3} \frac{1}{X_t} \le (\text {drift of } X_t) \le \frac{4(1+\varepsilon )}{3} \frac{1}{X_t} \end{aligned}$$

and hence we can couple $(X_t)_{t \in [\tau ,\tilde{\tau }]}$, where $\tau$ is any stopping time such that $X_\tau \le \varepsilon _1/2$ and $\tilde{\tau }=\inf \{t \ge \tau \,:\, X_t=\varepsilon _1\}$, to (scaled) Bessel processes $X_t^{\prime }$ and $X_t^{\prime \prime }$ which satisfy

118 $$\begin{aligned} \mathrm {d}X_t^{\prime }&= \frac{4}{\sqrt{3}} \mathrm {d}B_t + \frac{4(1-\varepsilon )}{3} \frac{\mathrm {d}t}{X_t^{\prime }} \end{aligned}$$

119 $$\begin{aligned} \mathrm {d}X_t^{\prime \prime }&= \frac{4}{\sqrt{3}} \mathrm {d}B_t + \frac{4(1+\varepsilon )}{3} \frac{\mathrm {d}t}{X_t^{\prime \prime }} \end{aligned}$$

and $X_\tau =X_\tau ^{\prime }=X_\tau ^{\prime \prime }$. Under this coupling $X_t^{\prime } \le X_t \le X_t^{\prime \prime }$ for any $t \in [\tau ,\tilde{\tau }]$.

Clearly

120 $$\begin{aligned} \{t \,:\, X_t^{\prime \prime }=0\} \subset \{t \,:\, X_t=0\} \subset \{t \,:\, X_t^{\prime }=0\} . \end{aligned}$$

By [18] Exercise XI.1.25 and [5] Theorem III.15, the Hausdorff dimension of the support of the local time of a Bessel process with index $\nu \in [-1,0]$ is $-\nu$. By Markov property of the Bessel process, the support of the local time is the entire set of times when the process is at the origin. Hence the set $\Sigma$ is sandwiched between two sets which have dimension arbitrarily close to 1 / 4. $\quad \square$

## Proof of Theorem 1.1

As a conclusion to this entire article we outline below the proof of Theorem 1.1.

As above, we consider a sequence of domains $\Omega _{\delta _n}$ converging to a domain $\Omega$ with respect to a fixed interior point $w_0$. Let $\phi _n$ be the conformal map from $\Omega _{\delta _n}$ to $\mathbb {D}$ normalized using $w_0$. Suppose also that we have a sequence of boundary points $v_{\delta _n} \in \partial \Omega _{\delta _n}$ that converge in the sense $\phi _n(v_{\delta _n})$ converges to a point on $\partial \mathbb {D}$. We use $v_{\delta _n}$ as the root point in the construction of the exploration tree.

The basic crossing estimates were established for the FK Ising exploration tree and its branches in Sect. 3.2, Theorem 3.4. Based on those estimates, the precompactness of probability laws of a single branch or a finite subtree (i.e., a subtree with a fixed number of target points) was shown in Sect. 3.7. By those results we can choose convergent subsequences. The structure of the tree is characterized by the target independence, the independence of the branches after disconnection and the martingale characterization of a single branch in Sect. 5. Here it is needed that the branches converge in the strong sense as capacity parameterized curves.

By these results, every sequence of finite subtrees of the approximating domains converges in distribution to a finite subtree of the $\hbox {SLE}(\kappa , \kappa -6)$ exploration tree with $\kappa =16/3$. The convergence takes place, for instance, in the unit disc $\mathbb {D}$ after a conformal transformation and under the metric defined in Sect. 3.3.1. In fact, it is possible to extend this convergence to the original domain (without the conformal transformation). See Corollary 1.8 in [14] for such a result.

The precompactness of the probability laws of the full tree and of the loop collection were established in Theorems 3.1 and 3.8, respectively. Therefore we can choose subsequences such that both the full tree and the loop collection converge. The above together with the finite-tree approximation in Theorem 3.6 implies that the full tree has a unique limit which is the $\hbox {SLE}(\kappa , \kappa -6)$ exploration tree with $\kappa =16/3$. Similarly using the finite-tree approximation in Theorem 3.9 we show that the loop collection has a unique limit which is characterized by the one-to-one correspondence to the exploration tree under the maps introduced in Sects. 1.2.1 and 2.3, see Theorem 3.11. This ends the outline of the proof. Notice that the convergence takes place in a topology where both the tree and the loop ensemble converge simultaneously and convergence occurs for the full objects, not just the finite tree approximations (with only a finite fixed number of target points).

Since the orientation of the image of the exploration tree when mapped to $\mathbb {D}$ depends on $\lim _n \phi _n(v_{\delta _n})$, while the scaling limit of the loop collection is the same for any sequence $v_{\delta _n}$. This gives the rotational invariance of the loop collection and thus implies the complete conformal invariance of the scaling limit.

## Appendix A. Distortion of Annuli Under Conformal Maps

Let $\Omega$ be a simply connected domain and $\phi : \mathbb {D}\rightarrow \Omega$ is a conformal map. The next two lemmas establish bounds for distortion of annuli in terms of their conformal modulus which is a constant times $\log (R/r)$ for an annulus $A=A(z_0,r,R)$. The proofs can be found from Section 6.3.3 of [13]. The results are needed in the proof of Theorem 3.1 above.

### Lemma A.1

(Distortion of annuli contained in $\mathbb {D}$). For any $\rho '>1$ sufficiently large, there exists $\rho >1$ independent of $\Omega$ and $\phi$ such that the following holds. Suppose that the annulus $A=A(z_0,r,R) \subset \mathbb {D}$ is such that $R/r > \rho$ and $R\le \frac{1}{2} (1-|z_0|)$. Then there exists an annulus $A^{\prime }=A(z_0^{\prime },r^{\prime },R^{\prime })$ with $R^{\prime }/r^{\prime } > \rho ^{\prime }$ such that $A^{\prime } \subset \phi (A)$ and $A^{\prime }$ separates the components of $\phi (A)$ in $\mathbb {C}$. Furthermore the dependency of $\rho$ and $\rho ^{\prime }$ can be made linear.

### Lemma A.2

(Distortion of annuli not fully contained in $\mathbb {D}$). For any $\rho ^{\prime }>1$ sufficiently large, there exists $\rho >1$ independent of $\Omega$ and $\phi$ such that the following holds. Suppose that the annulus $A=A(z_0,r,R)$ is such that $R/r > \rho$, $1-|z_0|<r$ and $R<1$ (that is, $\partial \mathbb {D}$ crosses *A*). Then there exists an annulus $A^{\prime }=A(z_0^{\prime },r^{\prime },R^{\prime })$ with $R^{\prime }/r^{\prime } > \rho ^{\prime }$ such that there exists a connected component *O* of $\Omega \cap A^{\prime }$ such that $O \subset \phi (A \cap \mathbb {D})$ and *O* separates the components of $\phi ((\partial A) \cap \mathbb {D})$ in $\Omega$.

## Appendix B. Auxiliary Results on a Priori Bounds

Denote conformal images of the FK Ising boundary loop exploration tree and the random cluster configuration under $\phi ^{-1}:\Omega \rightarrow \mathbb {D}$ by $\mathcal {T}_\mathbb {D}$ and $\omega _\mathbb {D}$, respectively. The following auxiliary results on the relation of the number of crossings by disjoint segments of tree branches and the number of disjoint open or dual-open crossings could be written in the original domain or any other “reference” domain. The unit disc is nice in the sense that the annulus has two sides on the boundary and thus the number $n-1$ appears in the formulation of the second lemma instead of a smaller number needed when the boundary makes more crossings of the annulus. The results are needed in the proof of Theorem 3.1 above.

### Lemma B.1

Suppose that $A=A(z_0,r,R) \subset \mathbb {D}$. If there are at least 2*n* disjoint segments of the tree $\mathcal {T}_\mathbb {D}$ crossing *A*, then there are at least *n* dual-open arms in the random cluster configuration $\omega _\mathbb {D}$.

### Proof

Any crossing of *A* by the tree $\mathcal {T}_\mathbb {D}$, when *A* is contained in the domain, is a subcurve of a single loop. Thus its left-hand side is entirely open and the right-hand side entirely dual-open. Due to topological reasons, any pair of adjacent minimal crossings (by the tree) have common type of boundary in the region between them. Thus in every second pair of adjacent minimal crossings have to have a dual-open crossing between them. $\quad \square$

### Lemma B.2

Suppose that $A=A(z_0,r,R)$ such that $B(z_0,r) \cap \partial \mathbb {D}\ne \emptyset$. If there are at least 2*n* disjoint segments of the tree $\mathcal {T}_\mathbb {D}$ crossing *A*, then there are at least $n-1$ dual-open arms in the random cluster configuration $\omega _\mathbb {D}$.

### Proof

Let *k* be the number of minimal crossings of *A* which touch the boundary so that their dual-open right-hand side touches free boundary (i.e. the vertex sets of the dual-open path and the dual boundary intersect) and that along each them there is at least one branching point. Then due to topological reasons *k* is at most 2. Let *m* be the total number crossings. Then $m-k$ crossings are disjoint from the boundary or touch the boundary by their open left-hand sides. In particular, for these types of crossings, the crossings consists only of a part following a single loop. Thus the entire right-hand side is dual-open. Similarly as in the previous proof, we see that there are at least $\lfloor (m-k)/2\rfloor$ dual-open crossings of *A*. $\quad \square$

## Appendix C. Auxiliary Results on Loewner Evolutions

The results of this section are needed in Sect. 5.4 above.

For a Loewner chain $(K_t)_{t \in [0,T]}$ in $\mathbb {H}$ driven by $(U_t)_{t \in [0,T]}$, let $(V_t)_{t \in [0,T]}$ be the function defined by

$$\begin{aligned} V_t = g_t( \sup ( K_t \cap \mathbb {R}) ) . \end{aligned}$$

The point $V_t$ is thus the image of the rightmost point on the hull under the conformal map $g_t$. The following lemma can be extracted from the proof of Proposition 3.12 in [23].

### Lemma C.1

For any $(U_t)_{t \in [0,T]}$ and $(V_t)_{t \in [0,T]}$ as above, it holds that $\int _0^T \mathbbm {1}_{V_t=U_t} \mathrm {d}t = 0$.

### Proposition C.2

Suppose that $\int _0^T \frac{\mathrm {d}t}{V_t - U_t} < \infty$. There exists a non-decreasing function $(\Lambda _t)_{t \in [0,T]}$ with $\Lambda _0=0$ such that

121 $$\begin{aligned} V_t = V_0 + \int _0^t \frac{2 \mathrm {d}s}{V_s - U_s} + \Lambda _t . \end{aligned}$$

### Proof

Let $\varepsilon >0$, $J_t^\varepsilon = \min \{ k \varepsilon > \sup ( K_t \cap \mathbb {R}) \,:\, k \in \mathbb {Z}\}$ and $\tilde{V}_t^\varepsilon = g_t(J_t^\varepsilon )$. Then it follows from monotonicity of $g_t$ that $V_t \le V_t^\varepsilon \le V_t + \varepsilon$. By the Loewner equation we can write

122 $$\begin{aligned} \tilde{V}_t^\varepsilon = \tilde{V}_0^\varepsilon + \int _0^t \frac{2 \mathrm {d}s}{\tilde{V}_s^\varepsilon - U_s} + \sum _{s \le t} \xi ^\varepsilon _s \end{aligned}$$

where $\xi ^\varepsilon _s \in [0,\varepsilon ]$ and $\xi ^\varepsilon _s \ne 0$ only when $\tilde{V}_u^\varepsilon - U_u$ hits zero as $u \nearrow s$. Thus the sum on the right-hand side is a finite sum. By Lemma C.1 and Lebesgue’s dominated convergence theorem the middle term in (122) converges to the integral in (121). Since also $\tilde{V}_t^\varepsilon$ converges uniformly to $V_t$, it holds that $\sum _{s \le t} \xi ^\varepsilon _s$ converges uniformly to some continuous function $\Lambda _t$ as $\varepsilon \rightarrow 0$. It follows that $\Lambda _t$ is non-decreasing and $\Lambda _0=0$. The claim follows. $\quad \square$

### Remark C.3

Notice that in fact, $\int _0^T \frac{\mathrm {d}t}{V_t - U_t} < \infty$ always. Namely,

$$\begin{aligned} \int _0^T \frac{\mathrm {d}t}{V_t - U_t + \varepsilon }&\le \int _0^T \frac{\mathrm {d}t}{\tilde{V}_t^\varepsilon - U_t} \le \frac{1}{2} \left( \tilde{V}_T^\varepsilon - \tilde{V}_0^\varepsilon \right) \le \frac{1}{2} \left( V_T - V_0 +\varepsilon \right) . \end{aligned}$$

Thus $\int _0^T \frac{\mathrm {d}t}{V_t - U_t} < \infty$ follows from Lebesgue’s monotone convergence theorem.
